# The interferon stimulated gene 20 protein (ISG20) is an innate defense antiviral factor that discriminates *self* versus *non-self* translation

**DOI:** 10.1371/journal.ppat.1008093

**Published:** 2019-10-10

**Authors:** Nannan Wu, Xuan-Nhi Nguyen, Li Wang, Romain Appourchaux, Chengfei Zhang, Baptiste Panthu, Henri Gruffat, Chloé Journo, Sandrine Alais, Juliang Qin, Na Zhang, Kevin Tartour, Frédéric Catez, Renaud Mahieux, Theophile Ohlmann, Mingyao Liu, Bing Du, Andrea Cimarelli

**Affiliations:** 1 CIRI, Centre International de Recherche en Infectiologie, Univ Lyon, Inserm, U1111, Université Claude Bernard Lyon 1, CNRS, UMR5308, ENS de Lyon, Lyon, France; 2 Institute of Biomedical Sciences and School of Life Sciences, East China Normal University, Shanghai, China; 3 Shanghai Emerging and Reemerging Infectious Disease Institute, Shanghai Public Health Clinical Center, Fudan University, Shanghai, China; 4 Université de Lyon, Université Claude Bernard Lyon 1, INSERM 1052, CNRS 5286, Centre Léon Bérard, Cancer Research Center of Lyon, Lyon, France; NYU School of Medicine, UNITED STATES

## Abstract

ISG20 is a broad spectrum antiviral protein thought to directly degrade viral RNA. However, this mechanism of inhibition remains controversial. Using the Vesicular Stomatitis Virus (VSV) as a model RNA virus, we show here that ISG20 interferes with viral replication by decreasing protein synthesis in the absence of RNA degradation. Importantly, we demonstrate that ISG20 exerts a translational control over a large panel of *non-self* RNA substrates including those originating from transfected DNA, while sparing endogenous transcripts. This activity correlates with the protein’s ability to localize in cytoplasmic processing bodies. Finally, these functions are conserved in the ISG20 murine ortholog, whose genetic ablation results in mice with increased susceptibility to viral infection. Overall, our results posit ISG20 as an important defense factor able to discriminate the *self*/*non-self* origins of the RNA through translation modulation.

## Author summary

The interferon-induced protein 20 (ISG20) is an RNA exonuclease endowed with broad antiviral properties. The prevailing mechanism of inhibition described for ISG20 indicates that this enzyme is capable of directly degrading viral RNA in the absence of apparent sequence specificity. This mode of action has been however challenged by recent studies that revealed that ISG20 could target specific structures on the hepatitis B virus, as well as by others that suggested inhibition in the absence of viral RNA degradation. We now demonstrate that ISG20 interferes with viral replication not by degrading viral RNA, but by impairing its translation. This mechanism of translational control targets all RNAs originated from ectopically introduced genetic material (through viral infection or transient transfection) that we define here collectively as *non-self*, independently from their viral/non-viral origins. However, ISG20 bears no effect on the translation of endogenous mRNAs transcripts, suggesting that ISG20 can discriminate between the cell’s own genetic material (*self*) and foreign one. By taking profit of their mode of replication through integration, or EBV-like episomal maintenance certain pathogens seemingly escape ISG20 by what can be defined as *self-mimicry*. Lastly, this mechanism of action is conserved in the ISG20 murine ortholog, whose genetic ablation results in mice with increased susceptibility to viral infection. Overall, our study reveals a novel role of ISG20 as a translational modulator of foreign genetic material playing important functions during viral infection *in vivo*.

## Introduction

ISG20 was identified in 1997 by the Mechti laboratory as a type-I interferon-induced protein associated to promyelocytic leukemia (PML) nuclear bodies and then to Nucleoli and Cajal bodies [1–3]. The protein belongs to the DnaQ-like (or DEDD, for Asp-Glu-Asp-Asp) 3’-5’ exonuclease superfamily that includes several enzymes with DNA or RNA specificities. Members of this superfamily share three conserved exonuclease motifs (Exo I, II and III) that surround the protein’s active site and contain the four DEDD residues important for metal ions chelation [4, 5]. ISG20 exhibits RNA, but no apparent DNA exonuclease activities [3, 6] and this property has been associated with inhibition of a broad range of RNA viruses such as *Flaviviridae* (Yellow Fever, West Nile, Dengue, Hepatitis C and Bovine Viral Diarrhea viruses), *Picornaviridae* (Hepatitis A virus), *Togaviridae* (Sindbis, Chikungunya and Venezuelan equine encephalitis viruses), *Rhabdoviridae* (Vesicular Stomatitis virus, VSV*)*, *Orthomyxoviridae* (Influenza virus), *Retroviridae* (Human Immunodeficiency Type 1 virus), *Hepadnaviridae* (Hepatitis B virus) and more recently several *Bunyaviridae* [3, 7–17], although certain RNA viruses do resist ISG20 as human Coronavirus and Phleboviruses [10, 17]. Direct viral RNA degradation by ISG20 has been proposed as the main mechanism of viral inhibition based on two main evidences: its potent exonuclease functions *in vitro* and the loss of antiviral effects observed upon the mutation of DEDD residues in the protein’s catalytic site, which constitute at present the only ISG20 mutants described in the literature. However, a number of studies have observed viral inhibition by ISG20 in the absence of viral RNA degradation [10, 11], raising the possibility that alternative mechanisms may be at play.

In this study, we determine that ISG20 acts by modulating mRNA translation and we provide evidence that this mechanism broadly targets mRNA transcripts originated from exogenous genetic material independently of its viral origins (that we globally refer to here as of *non-self* origins*)*, while sparing mRNAs of endogenous chromosomal origins (*self)*, or *self*-mimicking ones. Using VSV as a model for highly replicative RNA viruses, we show here that ISG20 decreases translation from a broad spectrum of viral as well as non-viral mRNAs, as long as they are ectopically expressed. While this mechanism of discrimination relies on an intact catalytic core, it takes place in the absence of target mRNA degradation and correlates with the ability of ISG20 to localize in cytoplasmic processing bodies (P bodies). The properties of human ISG20 are also conserved in its murine orthologue and through the CRISPR/Cas9-mediated generation of *isg20* knockout mice, we provide clear evidence that ISG20 plays an important role in the modulation of virus replication *in vivo*. Overall, our study reveals a novel role for ISG20 as a translational modulator capable of discriminating *self* from *non-self* mRNAs, offering novel perspectives on strategies of viral control and an appropriate animal model in which to test them.

## Results

### ISG20 inhibits VSV replication through a translational blockage

**Fig 1:**
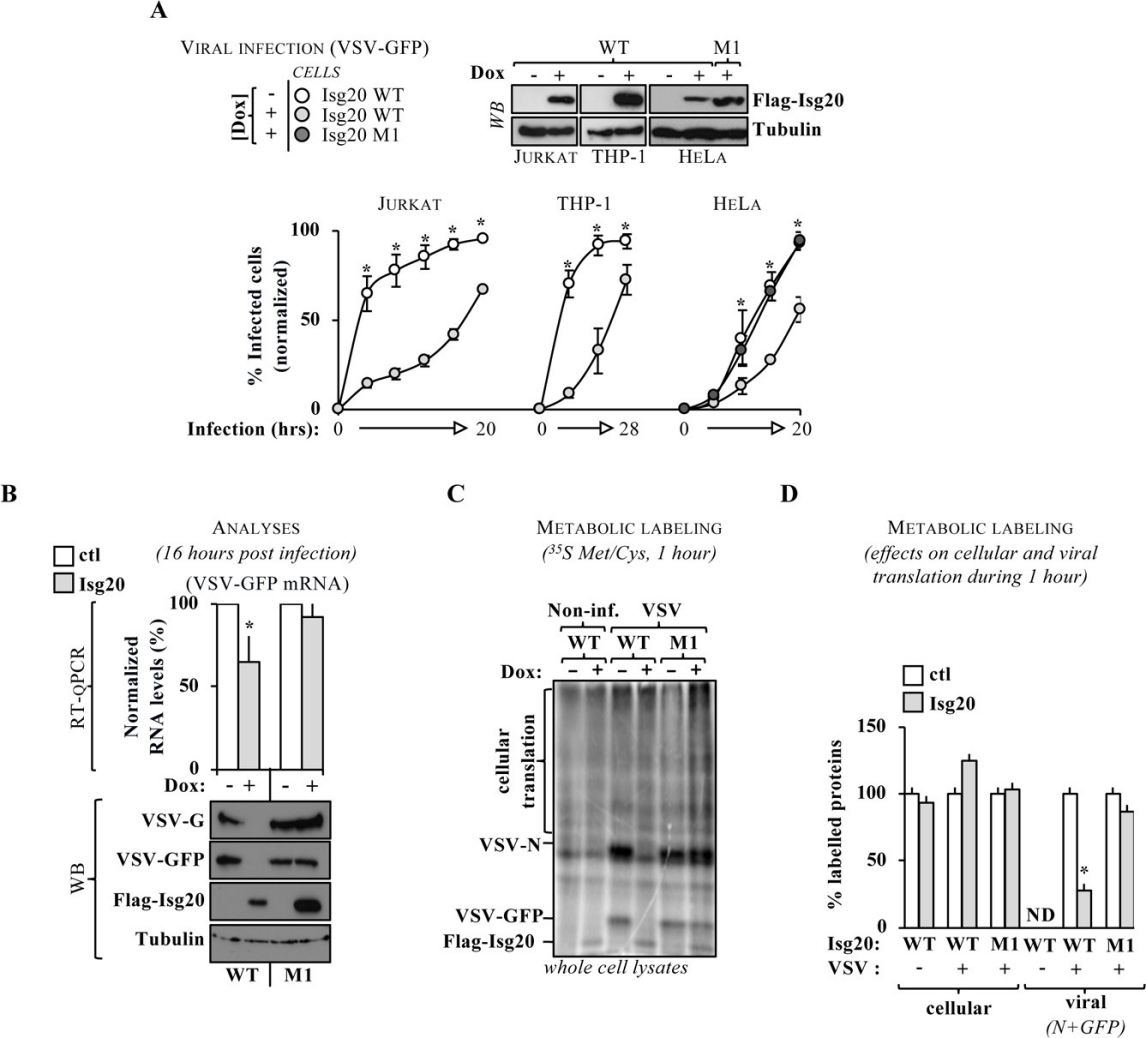
ISG20 inhibits VSV replication through a translational blockage. A) Dox-inducible ISG20 stable cell lines were generated upon retroviral-mediated gene transduction and selection. Twenty-four hours post-induction, cells were challenged with replication-competent VSV-GFP and the percentage of GFP-positive cells was determined at different time points post infection by flow cytometry (dox comprised between 0.5 and 1 μg/ml and multiplicities of infection, MOIs of 0.001 for Jurkat and THP-1 cells and of 0.01 for HeLa cells). B) Sixteen hours post infection, a fraction of HeLa cells expressing or not ISG20 were lysed and analyzed by RT-qPCR for the viral coded GFP RNA and by WB. C) As in B, but cells were incubated for one hour with ^35^S Met/Cys prior to cell lysis. Samples were then directly loaded onto an SDS-PAGE gel and analyzed by phosphor imager. D) Viral translation was quantified on the viral-coded proteins N and GFP, while cellular translation was quantified on the indicated portion of the gel (normalized to each no dox. condition). ND, not determined. The graphs present Means and SEM of two to four individual experiments, while the panels present typical results obtained. *; p≤0.05 between the indicated condition and control, according to a Student t test.

To apprehend the role/s of ISG20 during viral infection, dox-inducible Jurkat, THP-1 and HeLa cell lines stably expressing ISG20 were generated by retroviral-mediated gene transduction followed by the selection of the pool of transduced cells. ISG20 was then expressed at levels comparable to those observed in primary monocyte-derived macrophages and dendritic cells stimulated with IFNα, not only to keep within physiologically relevant boundaries, but also because we noted that higher expression levels of ISG20 were cytotoxic in established cell lines (**S1A and S1B Fig**). As such, all subsequent experiments were carried out at non-cytotoxic levels of ISG20 expression. Under these conditions, WT ISG20 inhibited VSV spread in the different cell lines tested, in contrast to a catalytically inactive ISG20 mutant (**Fig 1A**, M1, that corresponds to the already described D94 mutant, **S2 Fig** for infection with different viral multiplicities of infection, MOIs and **S3 Fig** for typical flow cytometry profiles and mean fluorescent intensities of GFP-positive cells). However, when HeLa cells were examined closely, we noted only a moderate reduction in the levels of viral-coded GFP RNA in ISG20-expressing cells in contrast to the drastic decrease in the amount of accumulated viral proteins by WB (**Fig 1B**, in this recombinant virus, *gfp* behaves as an additional transcriptional viral unit inserted between the M and the G genes, [18]). This was not due to loss of the RNA exonuclease abilities of ISG20, as these were readily measurable when ISG20 was immunoprecipitated from cell lysates and incubated with different exogenously-provided substrates (single or double stranded RNAs, as well as DNA:RNA hybrids, although not DNAs as expected [6], **S4A and S4B Fig**). This lack of specificity was not present in cells however, given that the ectopic expression of ISG20 did not lead to the indiscriminate degradation of cellular RNAs (ribosomal as well as small nucleolar RNAs previously shown to be associated to ISG20 [2]), nor of an Hepatitis C virus (HCV)-derived Luciferase reporter mRNA produced *in vitro* and then transfected into cells (**S4C and S4D Fig**), suggesting that in cells the RNase activity of ISG20 is tightly controlled.

Given that the protein accumulation defect was also observed after MG132-mediated inhibition of proteasome (**S5 Fig**), we decided to determine whether the effects of ISG20 related to translation inhibition. To this end, HeLa cells were metabolically labelled 16 hours post VSV infection for one hour with ^35^S methionine and cysteine, prior to cell lysis and phosphor imager analysis (**Fig 1C and 1D**). Under these experimental conditions, WT ISG20 imposed a strong translational inhibition to the expression of viral-coded proteins (N and GFP) in contrast to the catalytically inactive M1-ISG20 mutant. Cellular translation rates remained overall unperturbed, indicating that under these experimental conditions ISG20 was capable of discriminating viral from cellular mRNAs. Overall, these findings indicate that ISG20 may exert a novel antiviral inhibitory mechanism through the selective translational control of viral mRNAs.

### The antiviral activity of ISG20 strictly correlates with its ability to act as a general translational modulator of *non-self* genetic material

**Fig 2:**
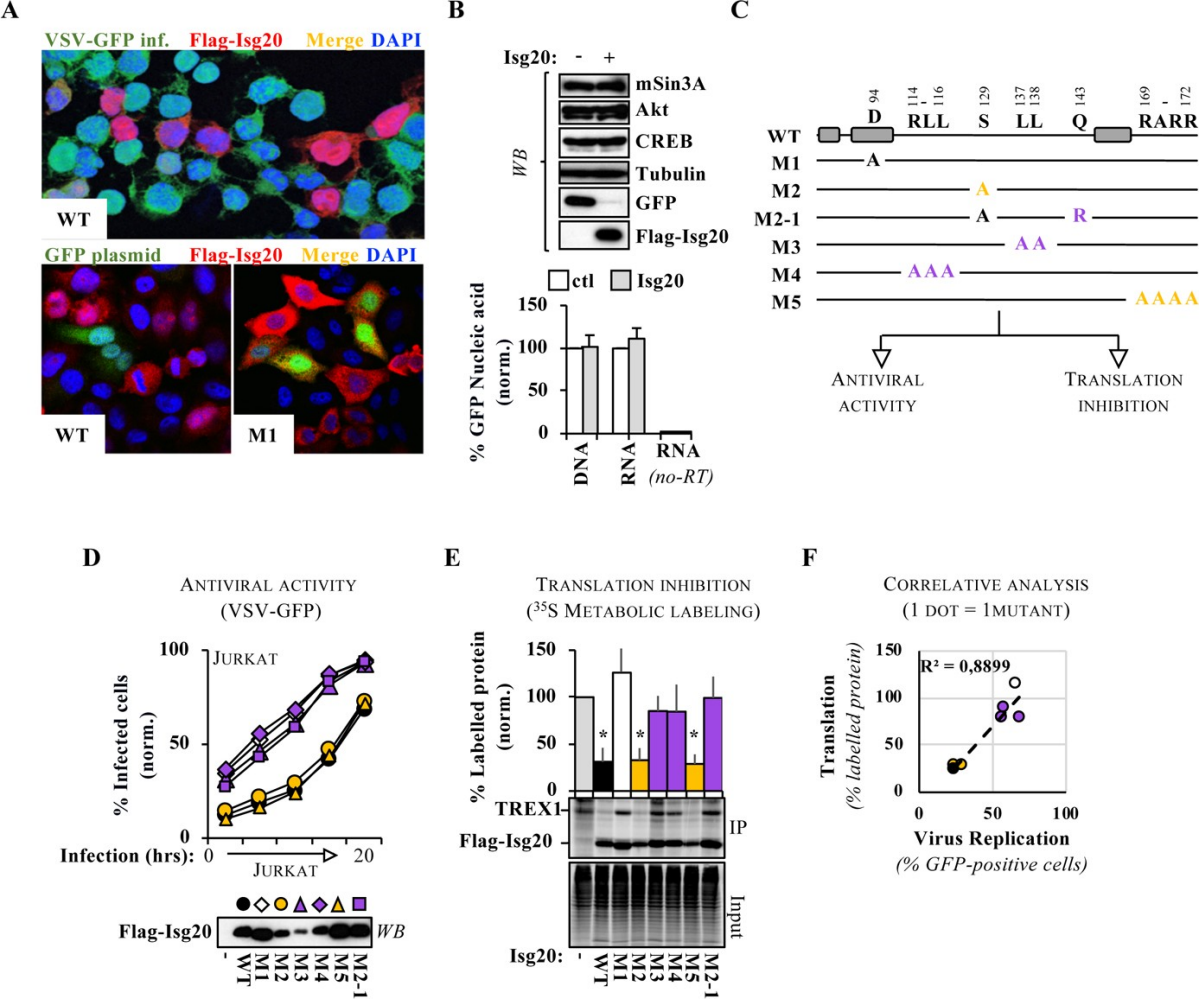
The antiviral properties of ISG20 strictly correlate with its ability to act as a general modulator of translation of *non self* genetic elements. A) HeLa cell lines stably expressing ISG20 were analyzed by confocal microscopy twenty-four hours after VSV-GFP challenge at an MOI of 0.01, or after transfection with DNA coding for GFP, as indicated. B) The same cells used in the bottom panels in A were also analyzed by WB, RT-qPCR (RNA), qPCR (DNA), as indicated. The pictures and the panels present typical patterns of expression, while the graph presents data obtained from four experiments. C) Schematic representation of the ISG20 mutants used here. The gray boxes indicate schematically the position of the three Exo domains of ISG20. The color code is used throughout the figure and refers to the antiviral activities of the indicated mutant: purple = lost; yellow = preserved. D) Dox-inducible ISG20 stable Jurkat T cells expressing the different mutants were obtained and infected with VSV, as specified above at an MOI of 0.001. The graph presents typical replication curves obtained out of three independent experiments. E) HEK293T cells were ectopically transfected with DNAs coding for ISG20 mutants and TREX1, used here as an additional reporter of ISG20 activity on ectopic gene expression. Cells were then metabolically labelled for one hour with ^35^S Met/Cys prior to cell lysis. A fraction of the soluble lysate was loaded to appreciate global translational effects (input), while the rest was used for immunoprecipitations with anti-flag antibodies specific for tagged ISG20 and TREX1 proteins. Samples were then loaded onto an SDS-PAGE gel for phosphor imager analysis. The graph presents Means and SEM of three to five independent experiments. *; p≤0.05 between the indicated mutant and control, according to a Student t test. F) Correlation between antiviral and translation inhibitory properties of individual ISG20 mutants.

To determine whether this mechanism of control could target more broadly non-*self* genetic material, we used transient DNA transfection which indeed represents a massive introduction of exogenous genetic material into the intracellular milieu. Transfection of ISG20-expressing HeLa cells with DNAs coding for a GFP reporter yielded a mutually exclusive pattern of expression upon confocal microscopy analysis, so that cells expressing ISG20 were rarely GFP-positive and vice versa, mirroring the results obtained during ongoing VSV infection (**Fig 2A**, for qualitative results). This pattern could be observed with WT ISG20, but not with the catalytically inactive M1 mutant and interestingly, loss of GFP protein accumulation was observed in the absence of effects on several endogenous proteins tested, as assessed by WB and in the absence of detectable changes in *gfp* DNA and mRNA levels (**Fig 2B**). Similar results were obtained with PCR amplicons placed at different location on the *gfp* mRNA and with reverse transcription reactions started with random hexamers or at the polyA tail with oligo-dT primers, excluding the possibility of partial end degradation of the *gfp* mRNA (**S6A Fig)**.

At the protein level, similar defects in GFP accumulation were observed in the presence of ISG20 both by WB using limiting dilutions of samples, as well as by FACS by adding defects in both the accumulation of GFP-positive cells and in their mean fluorescent intensity (**S6B and S6C Fig)**. Lastly, the inhibitory effect of ISG20 was dose-dependent (**S6D Fig)** and was observed for a large spectrum of ectopically expressed proteins independently from their DNA backbone, transfection method used or origins (viral or cellular genes, **S6E Fig)**.

Given the lack of described mutants in ISG20 outside its DEDD residues, a series of mutations were engineered in ISG20 outside the Exo domains, in sequences of potential interest (phosphorylation, sorting and nuclear import, through the ExPASy and the CBS protein sequence analysis prediction tools) to better apprehend the relationship between antiviral functions and translation control abilities. Not all generated mutants could be detected upon transient transfection and WB analysis, likely due to a compromised protein stability. However, five novel ISG20 mutants could be analyzed functionally (**Fig 2C)**. Although not pursued in depth in this study, these mutations could potentially affect phosphorylation on Serine (M2 and M2-1), or trafficking via mutations in dileucine domains (M4 and M3), or in nuclear localization signals (M5). Three ISG20 mutants lost their ability to inhibit VSV spread in culture (M3, M4 and M2-1, color-coded in purple), while two behaved as wild-type (M2 and M5, color-coded in yellow, **Fig 2D**, for replication over time in Jurkat T cells and **S7A Fig**, for a single time point in HeLa cells, 15 hours post infection).

The ability of these mutants to inhibit translation from ectopically expressed genes was assessed either directly by ^35^S metabolic labeling, or by WB (**Fig 2E** and S7B Fig, using TREX1 or GFP as examples of exogenous genes, respectively). Under these conditions, the M2 and M5 mutants behaved as WT and strongly reduced the rate of protein synthesis from the exogenous reporter genes, while in contrast the M3, M4 and M2-1 mutants behaved as the catalytically inactive M1-ISG20 and did not significantly affect protein translation, highlighting a near perfect correlation between antiviral abilities and translation inhibition of non-*self* material (**Fig 2F**). With the exception of the catalytically inactive M1, all mutants exhibited robust RNase activities *in vitro* **(S7C and S7D Fig**). Interestingly, two of the three mutations that altered the properties of ISG20 were spatially close and exposed at the surface of the crystal structure of ISG20 [5], potentially defining a region that may be involved either directly or indirectly in the regulation of the protein’s functions (**S7E Fig**).

### *Self* mimicry allows the escape of target genes’ mRNAs from the effects of ISG20

**Fig 3:**
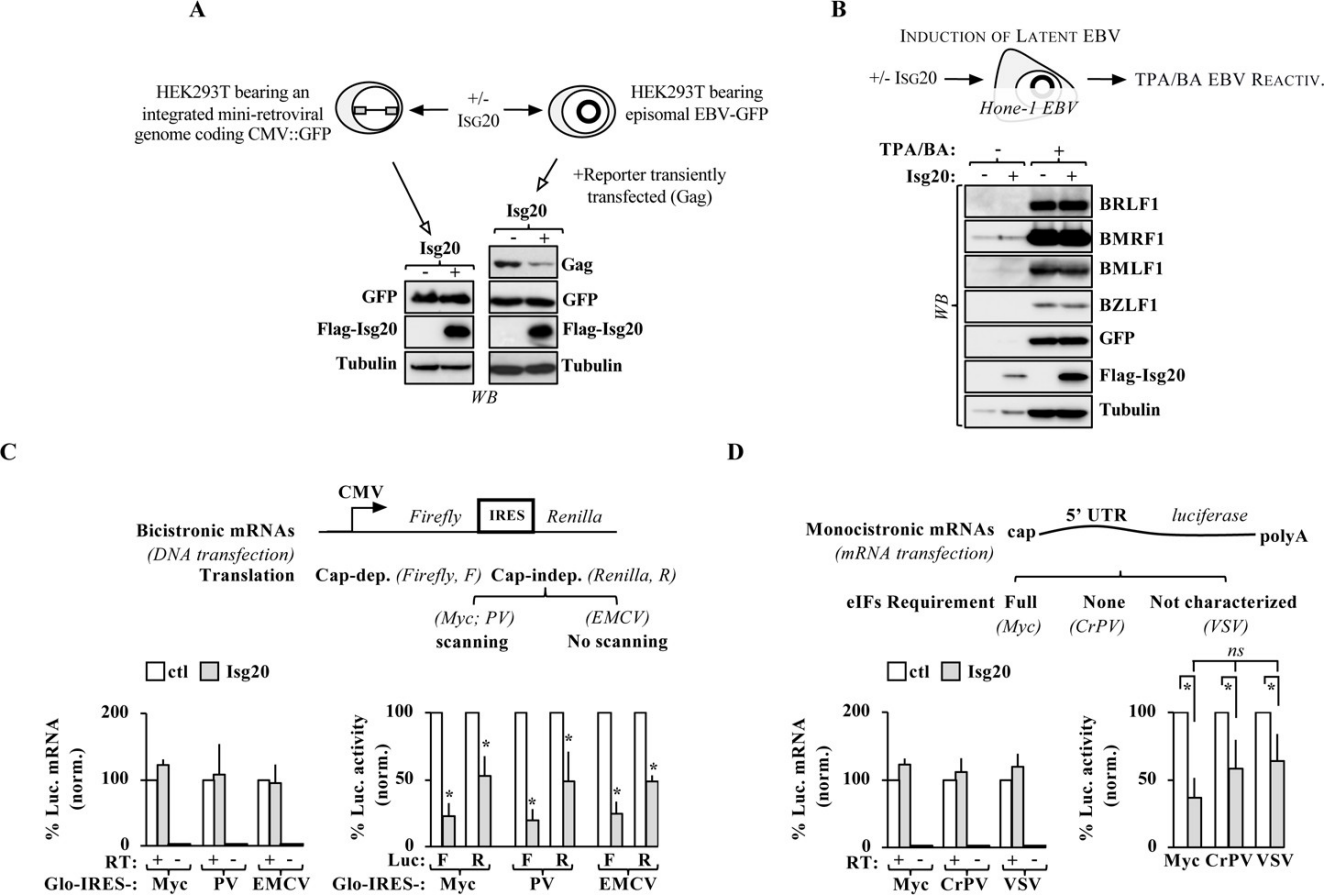
Gene mimicry of *self* through integration into the host genome, or EBV-mediated episomal maintenance, allows escape from ISG20 translational targeting. A) HEK293T cells were first stably transduced with a retroviral-based vector bearing a CMV-GFP expression cassette and then transiently transfected with an ISG20-expressing DNA vector. Cells were analyzed forty-eight hours after by WB. Similarly, HEK293T cells carrying a stable EBV-GFP episomal genome were transiently transfected with DNAs coding for ISG20 and HIV-1 Gag, used here as a reporter of the activity of ISG20 on ectopic expression. B) Hone-1 cells that bear a stable latent episomal EBV genome were transfected with ISG20 prior to stimulation with 12-*O*-tetradecanoylphorbol-13-acetate (TPA) and butyric acid (BA) that reactivates the life cycle of EBV via a PKC-dependent mechanism. Cells were lysed twenty-four hours later and analyzed by WB for the indicated EBV proteins. Panels present typical results obtained. C) HEK293T cells were transfected with ISG20 along with the indicated bicistronic DNA vectors. The first cistron bears the 5’UTR of the cellular gene globin (Glo). The second cistron is under the control of several IRESes derived from the cellular gene myc (Myc), polyomavirus (PV), or the encephalomyocarditis virus (EMCV). mRNA levels have been normalized to controls (no ISG20 condition) and luciferase activities are reported after mRNA normalization. D) Translation-competent mRNA generated *in vitro* were transfected in ISG20 expressing cells, prior to cell lysis one hour afterwards and analyses by RT-qPCR or luciferase assays. The 5’UTRs of these mRNAs varied with respect to the requirement of specific eIFs and were derived from the cellular gene myc (Myc), the cricket paralysis virus (CrPV) and VSV. The graphs present Means and SEM of 3 (monocistronic) and 8 (bicistronic) independent experiments. *; p≤0.05 between control and ISG20, as indicated, according to a Student t test.

VSV infection and ectopic DNA or RNA transfection do not bear much in common apart from the fact that both represent artificial injections into the cell of *non-self* genetic material from without. To determine whether ISG20 could be an IFN-induced mechanism of defense acting at the translational level, we determined the effects of ISG20 on exogenous expression cassettes that mimicked cellular host genes. To this end, retroviral-mediated gene transduction was used to stably integrate into the host genome the identical CMV-GFP expression cassette used in Fig 2A. After a three weeks selection, cells were transiently transfected with WT ISG20, lysed and analyzed by WB (**Fig 3A**). In this case, ISG20 did not modify the amount of produced GFP, indicating that, when expressed from the cell’s genome, the GFP-coding mRNA is ISG20-resistant and suggesting that cellular mRNAs mimicry (meant here as the ability to behave as, or similarly to, cellular genes) could constitute an escape mechanism from ISG20.

Some DNA viruses as the Epstein-Barr virus (EBV, *Herpesviridae*) can be stably maintained in an extrachromosomal latent form in the nucleus of target cells (referred to as episomes) using the cellular DNA machinery for their duplication and segregation into dividing cells. Although both transiently transfected DNA and the EBV genome are extrachromosomal, only the latter presents a chromatin status identical to the host genome [19, 20]. To determine whether this, rather than integration *per se*, could be a determining factor in the susceptibility to ISG20, HEK293T cells bearing stable EBV-GFP episomes were transiently transfected with DNAs coding for WT ISG20, in addition to a control plasmid coding for HIV-1 Gag (that served as a control for *non-self* genetic material within the same cells, **Fig 3A**). Upon cell lysis and WB analysis, ISG20 did not affect the amount of GFP expressed from the EBV genome, whereas it did affect the amount of Gag. To further strengthen this point, the effect of ISG20 on EBV infection was determined in Hone-1 cells upon the reactivation of the viral lytic cycle with TPA/BA (**Fig 3B**) [21]. ISG20 did not affect the production of several EBV proteins that typically initiate its lytic cycle indicating that EBV mRNAs were, as cellular ones, largely resistant to the action of ISG20. Also, ISG20 did not modify the extent of EBV infection, when virion particles produced under these conditions were used to challenge Raji cells **(S8 Fig**).

Overall, these results indicate that ISG20 discriminates mRNAs based on their *self/non-self* genesis and also underline the fact that certain viruses, as EBV, may have devised mechanisms to resist this inhibition due to their specific modes of replication.

### ISG20 does not act by influencing the nucleo-cytoplasmic transport of mRNAs and does not affect translation initiation

To determine whether ISG20 could affect translation through modifications in either splicing or transport of mRNAs from the nucleus to the cytoplasm, its activity on intron- or intronless-luciferase based constructs was assessed [22] and the accumulation of target mRNAs was determined in cytoplasmic and nuclear fractions (**S9A and S9B Fig**). ISG20 exerted similar inhibitory effects on both intron and intronless constructs and it did not appreciably modify the distribution of target mRNAs in cytoplasm and nucleus, overall suggesting that ISG20 does not affect the nucleo-cytoplasmic transport of mRNAs. Lastly, ISG20 could not be detected in crude ribosomal fractions, indicating a lack of stable association with ribosomes **(S9C Fig**).

A series of constructs was used to determine whether ISG20 affected translation initiation by comparing more specifically its effects on cap-dependent versus internal ribosomal entry site (IRES)-translation (**Fig 3C**, through the use of bicistronic vectors), as well as its effects on RNAs with different 5’ untranslated regions (5’ UTRs) that require or not eIF factors to initiate translation (**Fig 3D**).

ISG20 was capable of inhibiting both cap- and IRES-dependent translation from bicistronic mRNAs in the absence of mRNA degradation and interestingly this occurred on IRES sequences in which translation initiation required ribosomal scanning along the mRNA or not (**Fig 3C,** IRESes obtained from c-Myc, polyomavirus-PV, or the encephalomyocarditis virus-EMCV, respectively).

To further determine whether ISG20 acted on translation initiation and more specifically through specific initiation factors (eIFs), capped and polyadenylated mRNAs coding luciferase under the control of several 5’ untranslated regions (5’ UTRs) were generated *in vitro* and directly transfected in ISG20-expressing cells (**Fig 3D).** Under these conditions, ISG20 was able to inhibit translation of target RNAs containing either the 5’ UTR of c-Myc, that requires the complete set of eIFs, or of the cricket paralysis virus (CrPV) that requires none in the absence of mRNA degradation. We noted that in the case of direct mRNA transfection the extent of ISG20 inhibition was lower than in the case of DNA transfections, but we believe this is mainly due to the shorter time frame elapsing between transfection and luciferase assay (one hour, as opposed to overnight). As expected, the catalytically inactive M1-ISG20 mutant lost its ability to inhibit translation from these reporter constructs **(S10 Fig**).

Overall, these analyses underline the fact that the translational interference of ISG20 proceeds in the absence of overt mRNA degradation in a manner that appears independent from the presence of specific initiation factors during 5’ cap loading.

### IFIT1 does not participate in ISG20-mediated translation inhibition

**Fig 4:**
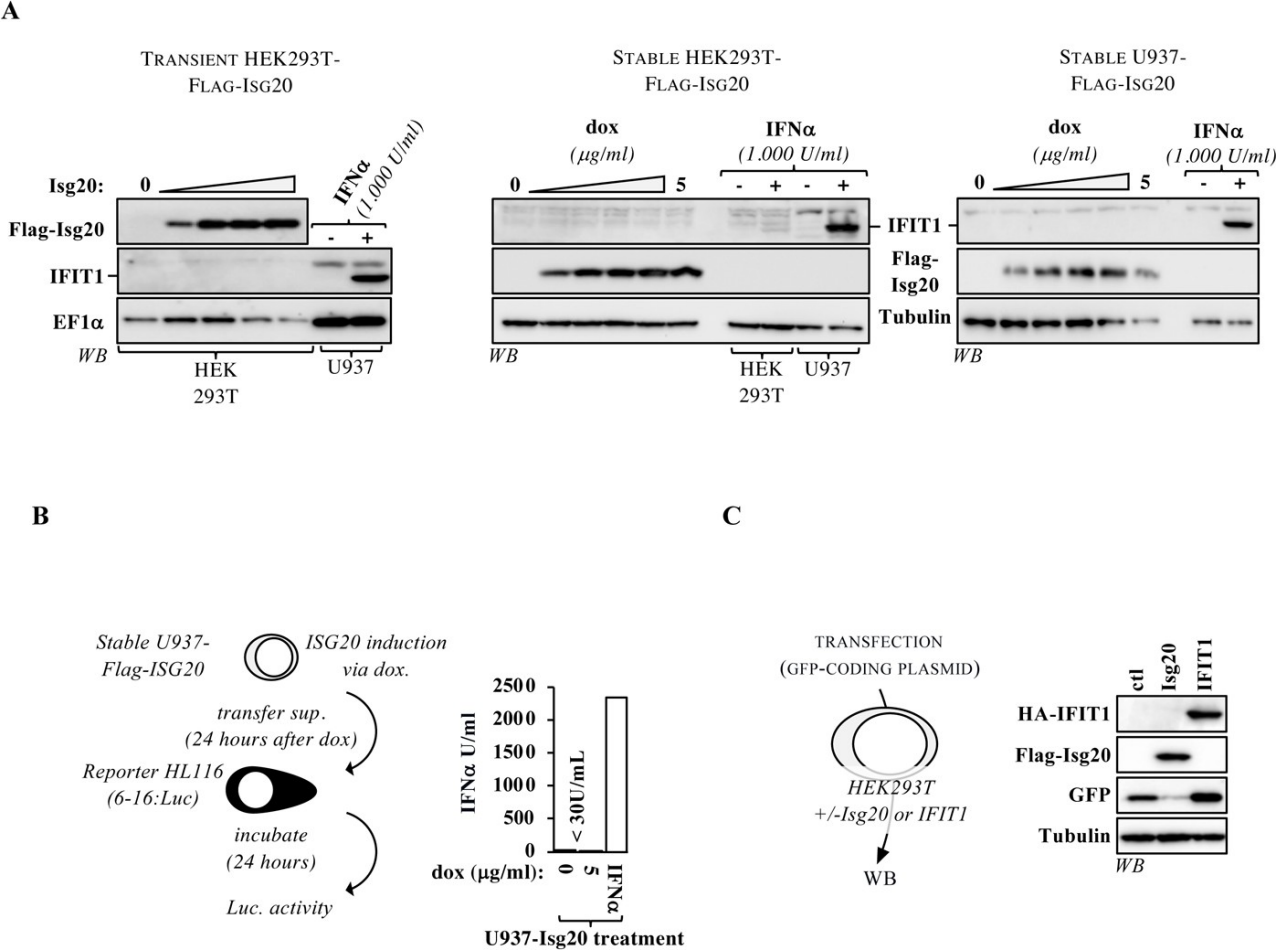
ISG20 does not induce IFIT1 expression and IFIT1 is unable to block expression from ectopically transfected (*non self)* genetic material. A) HEK293T cells that had been transiently transfected with increasing doses of ISG20 along with a fixed dose of GFP were analyzed by WB to assess the IFIT1 expression levels. The samples used for this experiment are the ones presented in S6D Fig to determine the dose-dependent effect of ISG20 on GFP. The panel presenting ISG20 levels is thus a duplication of the one of S6D Fig and is reproduced here only for clarity’s sake. In parallel, IFIT1 expression levels were analyzed by WB in HEK293T and U937 stable-ISG20 cells stimulated for twenty-four hours with different concentrations of doxycycline, or with IFNα2 (1.000 U/ml). B) To measure the possible presence of IFN subtypes in the supernatant of cells expressing ISG20, U937 cells that express the most readily detectable levels of IFIT1 were stimulated as indicated and the supernatant was then transferred to reporter cells expressing Luciferase under the control of the IFN-inducible 6–16 promoter. Luciferase production from reporter cells was then determined twenty-four hours later. The linearity and limit of detection of the assay (30 U/mL in our hands) are routinely determined with external dilutions of IFN. C) HEK293T cells were transiently transfected with plasmids coding for either ISG20 or IFIT1 along with a GFP-coding plasmid followed by WB analysis. The graph present results obtained from 2 independent experiments, while the WB panels display representative results obtained from 3 independent experiments.

When expressed in two clonal mouse embryonic fibroblast (MEF) cell lines, ISG20 has been recently described to drive type I interferon responses resulting in the expression of different interferon-sensitive genes among which the interferon-induced protein with tetratricopeptide repeats 1 (IFIT1) [11]. IFIT1 recognizes viral uncapped RNAs (often defined as *non-self*, although the definition used in our study includes more broadly all foreign genetic elements) and has been extensively associated to translation inhibition [23–26], this work raised the possibility that ISG20 could inhibit translation indirectly via the induction of IFIT1. To address this possibility, we analyzed IFIT1 expression in both ectopically transfected HEK293T cells, as well as in stable HEK293T and U937 cells, a myeloid cell line highly sensitive to IFN (**Fig 4A**). When HEK293T cells were ectopically transfected with increasing amounts of ISG20-coding DNAs, IFIT1 remained undetectable unless IFNα was provided (1.000 U/ml). Similarly, ISG20 induction by doxycycline in stable HEK293T and U937 cells did not induce a concomitant IFIT1 production, indicating that ISG20 does not induce IFIT1 expression under the experimental conditions used here. To more generally appreciate whether ISG20 induced an IFN program, we measured the levels of type-I IFNs potentially secreted upon induction of ISG20 in the supernatants of U937 cells that are more responsive to IFN stimulation than HEK293T. To this end, ISG20 expression was induced via doxycycline in stable U937 cells and 24 hours later supernatants were harvested and incubated with reporter HEK293T cells bearing an integrated expression cassette responsive to type I interferons (6–16:Luc). Reporter cells were then lysed twenty-four hours after and luciferase measured. The levels of type-I IFN measured via this assay remained below the threshold of detection (30 U/mL, in our hands), indicating the absence of major IFN up-regulation following ISG20 induction (**Fig 4B**).

Lastly, we wished to determine whether IFIT1 could exert similar activities than ISG20. To this end, HEK293T cells were transiently transfected with DNA coding these proteins along with GFP as *non self* genetic material (**Fig 4C**). Under these conditions, ISG20 but not IFIT1 inhibited the accumulation of GFP, while both inhibited VSV infection (**S11 Fig**). IFIT1 has been described to act in complex with other IFIT proteins [27]. Given that its ectopic expression alone is sufficient to inhibit VSV replication, we believe that other IFITs are expressed at a basal level under the conditions used here and that IFIT1 may be a limiting component of the complex. On the whole, our results indicate that IFIT1 is not involved in the mechanism of translation inhibition by ISG20 given that: 1) ISG20 does not induce the expression of IFIT1, at least under the experimental conditions used in this study and 2) that ISG20, but not IFIT1, is capable of inhibition of translation from *non-self* genetic material.

### ISG20 inhibition of translation correlates with its association with cytoplasmic P bodies

ISG20 is largely distributed throughout the entire cell [3, 10], although certain studies reported a more specific association with nuclear bodies (PML nuclear bodies, nucleoli and Cajal bodies [1, 2]). To determine whether this was the case, we employed a commonly used technique to reveal protein association to nuclear bodies. Cells were transiently transfected in duplicate with ISG20 (transfection rates ≥80%) and while one sample was immediately fixed, the other was previously treated with detergent prior to fixation to reveal proteins strongly associated to nuclear bodies. Under these conditions, partial co-localization of ISG20 was observed with splicing speckles (SC35 nuclear bodies, **S12A Fig**), but the signal was essentially lost upon detergent pre-extraction, suggesting that the association between ISG20 and NBs may be very weak.

**Fig 5:**
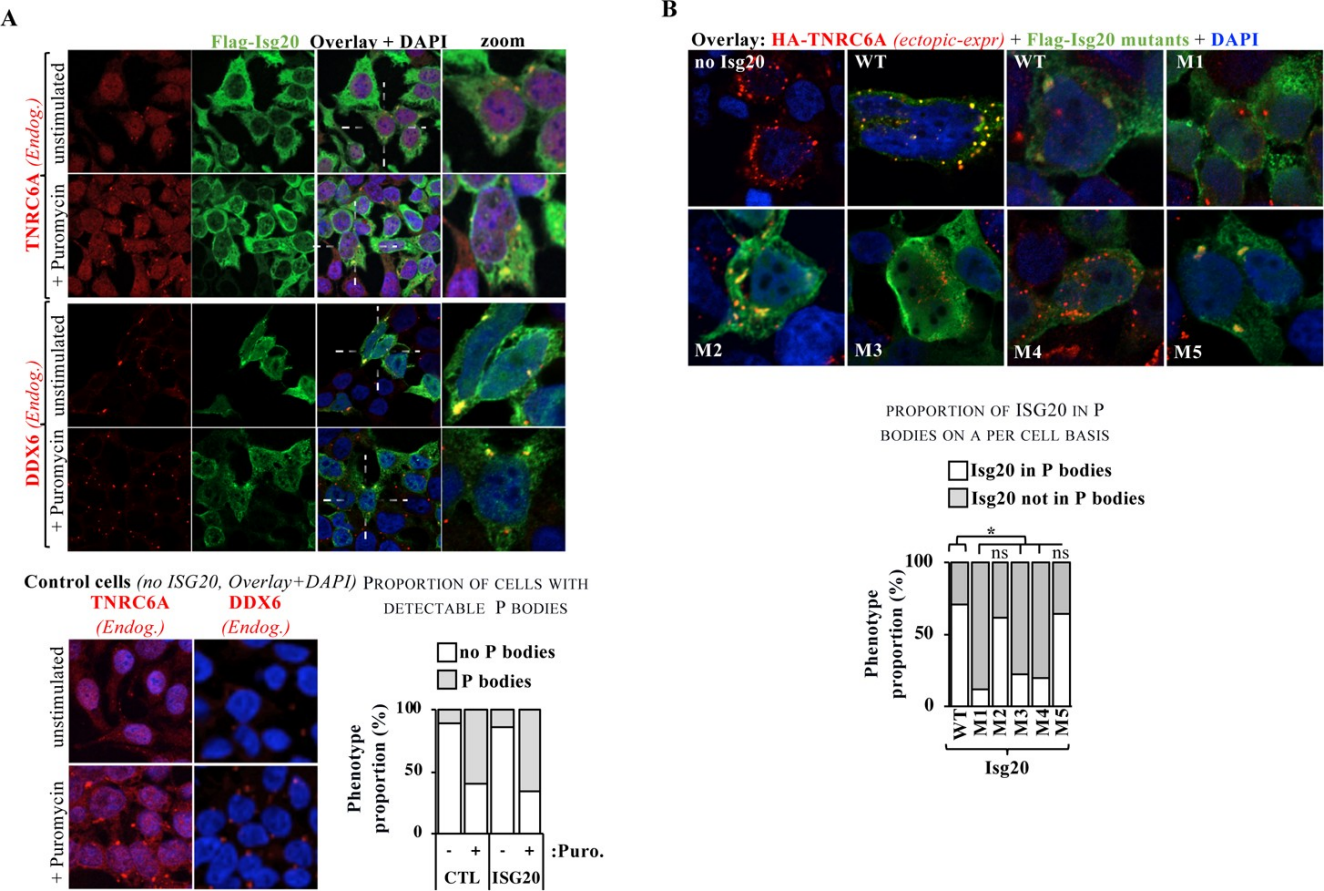
ISG20 co-localizes with P bodies. A) HEK293T ISG20-expressing cells were analyzed by confocal microscopy along with two markers of P bodies, TNRC6A and DDX6 in the presence or absence of a one-hour incubation with 100 μg/ml of Puromycin that induces a translational stress known to increase the number of cells expressing P bodies as well as the number of P bodies on a per cell basis [45]. Control cells not expressing ISG20 are shown only as a zoomed overlay, while the complete panel are presented in S12B Fig. The graph presents the proportion of cells exhibiting detectable P bodies in the presence or absence of puromycin and/or ISG20. B) The different ISG20 mutants were similarly analyzed by confocal microscopy with the exception that an HA-tagged, destabilized form of TNRC6A was also concomitantly expressed by transfection to increase the extent of P bodies accumulation. Representative pictures and distributions (out of >100 cells per condition/mutant in two to three independent experiments) are shown. The graph presents the proportion of P bodies with or without ISG20 in double-positive cells.

Instead, we noted a partial colocalization between ISG20 and both the endogenous forms of the trinucleotide repeat containing 6A protein (TNRC6A) and the DEAD-box helicase 6 (DDX6), two well-established markers of P bodies [28, 29] (**Fig 5A** for HEK293T cells expressing ISG20 and **S12B Fig** for the complete panels of control cells) both in unstimulated cells, as well as in cells treated for one hour with Puromycin, drug that induces translational stress and increases both the proportion of cells exhibiting P bodies, as well as their number on a per cell basis. However, ISG20 did not affect the proportion of cells presenting or not P bodies over controls, suggesting that ISG20 does not induce P bodies by itself.

To study the correlation between the antiviral phenotype of ISG20 and P bodies, HEK293T cells expressing the different ISG20 mutants were examined by confocal microscopy after ectopic expression of a destabilized version of TNRC6A, that allows for an easier visualization of P bodies (**Fig 5B** for representative pictures and graph for comprehensive quantification of the proportion of P bodies within cells that scored positive for ISG20). Under these conditions, WT ISG20 was present in 70% of P bodies in double-positive cells, a proportion similar to the functional ISG20 M2 and M5 mutants (62% and 64%, respectively). In contrast, the proportion of ISG20 present in P bodies dropped substantially in all the non-functional mutants examined (M1, M3 and M4, with rates of 12%, 22% and 19%, respectively). Thus, these results indicate that P bodies and/or P bodies localization may be critical for translation inhibition and antiviral properties of ISG20.

### Murine ISG20 presents the main functions of its human orthologue

**Fig 6:**
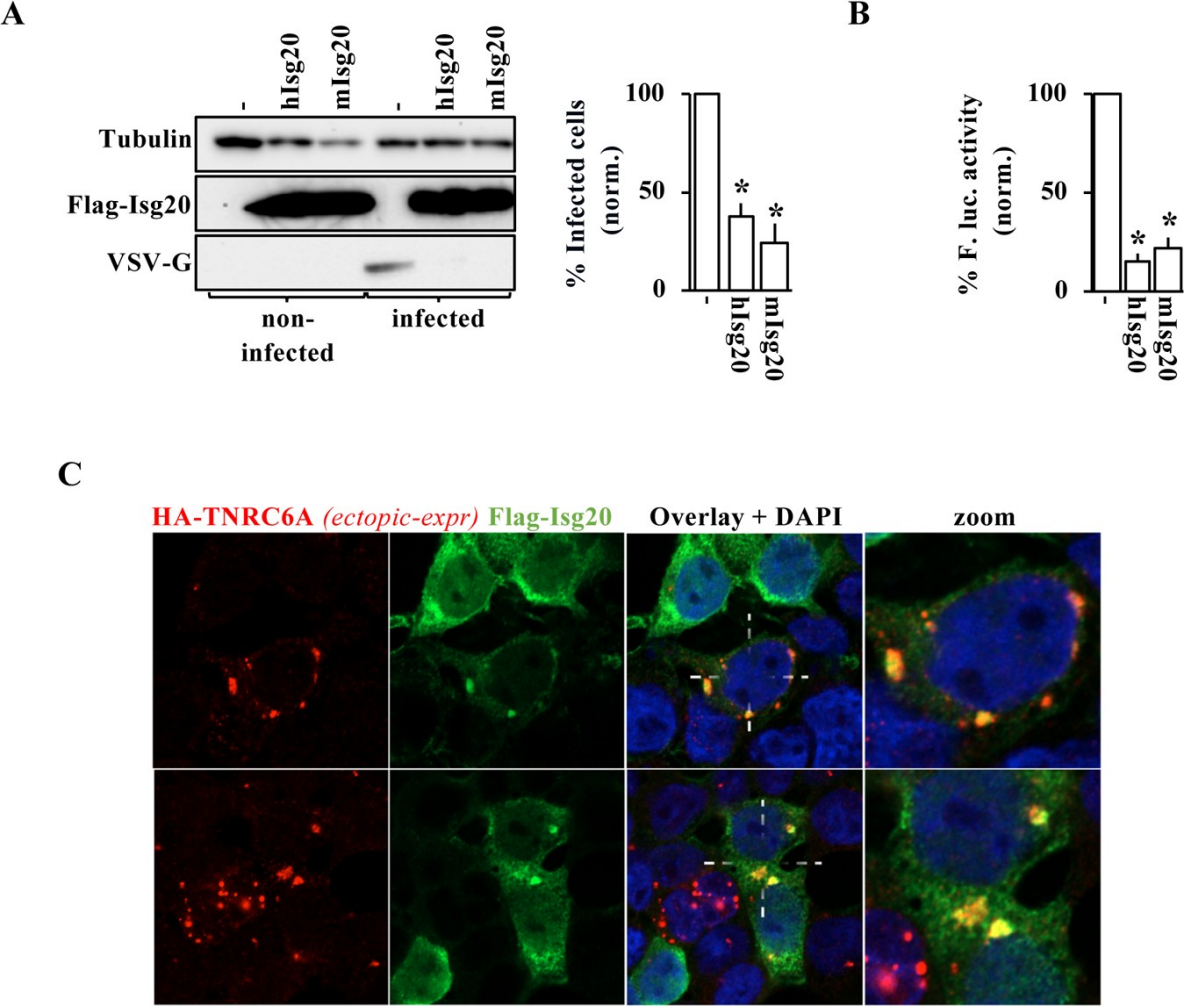
Murine ISG20 recapitulates the main functions described here for its human orthologue. A) Murine ISG20 was ectopically expressed inHEK293T cells prior to VSV challenge at an MOI of 0.1. The extent of infection was monitored by WB (panels) or FACS (graph) twenty-four hours post infection. B) as in A but cells were transiently transfected with DNA coding a Firefly luciferase reporter and the accumulation of luciferase in cell lysates was evaluated one day after. C) as in A but cells were also co-transfected with TNRC6A that allows for an easier visualization of P bodies. The graphs present Means and SEM of three independent experiments, while panels and pictures present representative results obtained. *, p< 0.05 according to a Student t test.

Murine and human ISG20 are highly conserved and display more than 80% identity at the amino acidic level (**S13 Fig**) and a recent report indicated that murine ISG20 was also endowed with anti-viral properties [11]. To determine whether the remaining functions of ISG20 described here were also similarly conserved, murine ISG20 (mISG20) was ectopically expressed in HEK293T cells and compared to its human counterpart (hISG20). Under these conditions, mISG20 was able to inhibit VSV spread through the culture (**Fig 6A**), it impaired expression from transfected DNA coding for a luciferase reporter (**Fig 6B**) and was also able to cluster along with TNRC6A bodies (**Fig 6C**). Therefore, these results indicate that the key functions of ISG20 described here are mainly conserved between these two animal species.

### ISG20 is important for viral control *in vivo*

**Fig 7:**
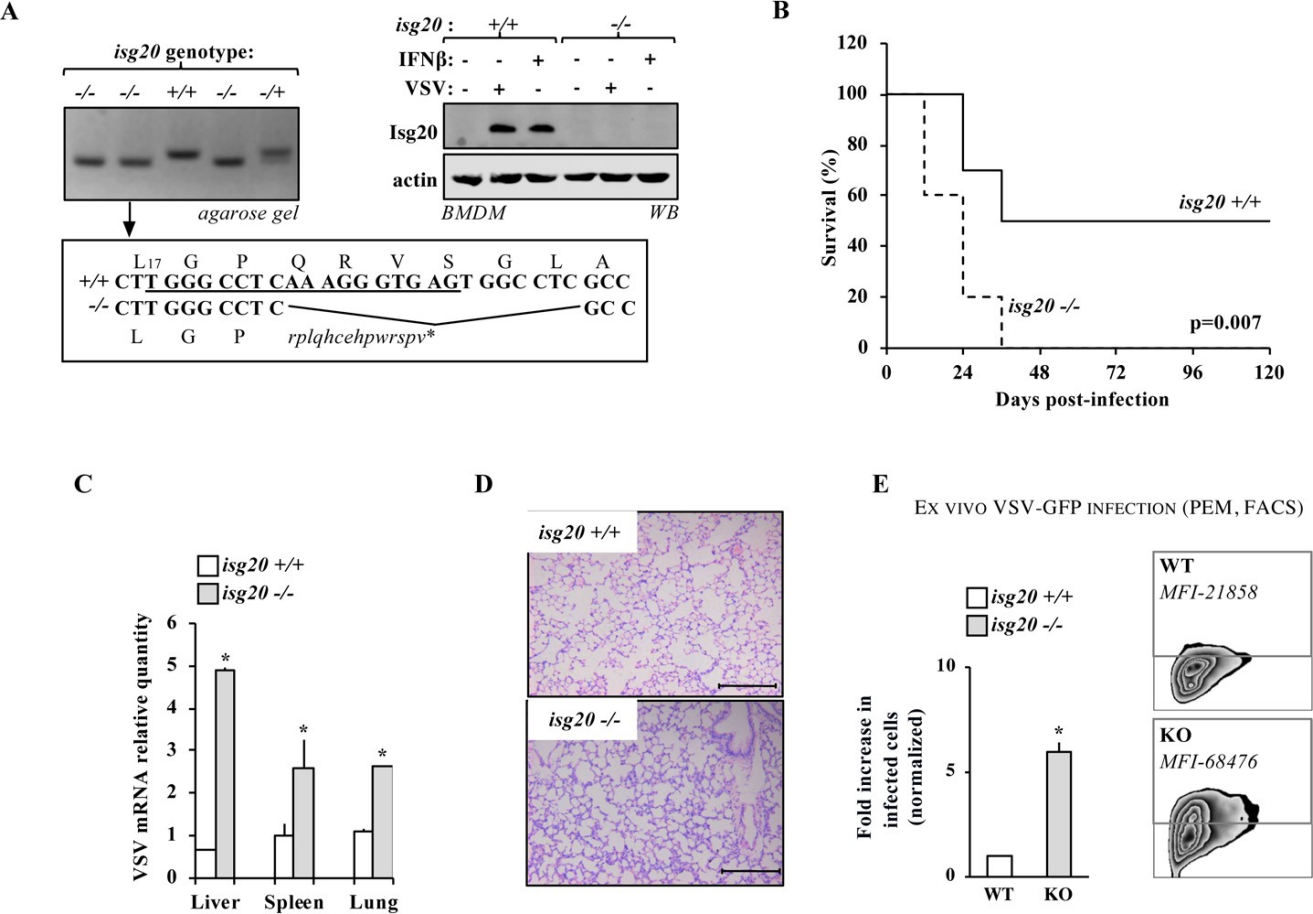
*Isg20* -/- mice exhibit higher susceptibility to VSV infection. A) CRISPR/Cas9 was used to generate a functional ISG20 knockout mouse. The target sequence is underlined and the resulting indel mutation and downstream open reading frame, as well as corresponding PCR in founders, are shown. To detect ISG20 protein, bone marrow-derived macrophages were stimulated either with IFN β or with VSV for eight to twelve hours prior to WB analysis. B) *isg20*-/- and +/+ age- and sex-matched littermates were intraperitoneally infected with VSV. The graph presents survival rates of each group. C) The extent of VSV replication in the indicated tissues was determined by RT-qPCR. D) Representative lung sections are shown upon hematoxylin and eosin staining that preferentially label inflammatory cells. E) Peritoneal macrophages (PEMs) were prepared from *isg20* -/- or control mice and then challenged *ex vivo* with VSV-GFP (MOI 0.5) prior to flow cytometry analysis twenty-four hours later. A representative zebra plot is shown with respective MFI of GFP-positive cells. The graphs present Means and SEM of three independent experiments (20 animals in two independent experiments in survival experiments).

To determine the importance of ISG20 during viral control *in vivo*, *isg20* knockout mice were generated by CRISPR mediated gene editing (**Fig 7A**, left panel) and the functional ablation of ISG20 was controlled upon *in vitro* stimulation of Bone-Marrow derived Macrophages (BMDM, **Fig 7A**, right panel). Isg20-/- mice were viable and displayed the expected Mendelian frequency. When animals were challenged intraperitoneally with VSV, the survival rates of *isg20*-/- mice were severely compromised with respect to age- and sex-matched control groups (**Fig 7B**), indicating that ISG20 plays an important controlling role during viral containment *in vivo*. The higher replicative capacity of VSV was evident in different organs and correlated with higher infiltration of inflammatory cells as observed upon hematoxylin and eosin staining (**Fig 7C and 7D**, lung sections). As expected, peritoneal macrophages (PEM) isolated from *isg20*-/- mice exhibited an increased susceptibility to infection (**Fig 7E**, in both percentage of infected cells as well as in their MFI), thus confirming a primary role for ISG20 in intracellular innate defenses. Overall, these results indicate that ISG20 is an important contributor of viral replication control *in vivo*, corroborating similar results obtained in a previously published animal model [11].

## Discussion

In this study, we determine that ISG20 acts as an important antiviral factor *in vitro*, as well as *in vivo*, given that mice genetically deficient in ISG20 exhibit higher mortality following viral challenge. The antiviral properties of ISG20 are not linked to its ability to directly degrade viral RNA, but rather to its capacity to interfere with the process of translation. This mechanism of translational regulation targets and is able to distinguish viral RNAs as well as RNA originated from ectopically transfected DNA (that we globally refer to as *non-self*, for mRNAs originated from *non-self* genetic elements) from cellular mRNAs (or *self*) that are instead resistant to ISG20, under the experimental conditions used here. Finally, we determine that resistance to ISG20 can be achieved via integration into the host genome, or through EBV-like maintenance in stable episomes through what can be defined as *self* mimicry.

ISG20 is a potent and non-specific RNA exonuclease *in vitro* and this property had long been hypothesized to underpin a broad mechanism of inhibition via the direct degradation of the RNA genome of a large spectrum of RNA viruses, albeit not all [2, 3, 6–17]. However, a few studies failed to observe a decline in viral RNA levels in the presence of ISG20 [10, 11] and evidence of direct binding of target RNAs to ISG20 in cells is lacking. The data presented in our study also argue against overt degradation of viral RNA and point instead to an interference with the process of translation that not only targets viral RNAs, but more generally distinguishes mRNAs according to their *self/non-self* origins (according to the broader definition used in this study). Despite the fact that the catalytically inactive mutant M1 loses both its exonuclease and translation inhibition activities, whether the former is required for translation inhibition remains unclear. Indeed, mutations in the DEDD residues may potentially exert multiple effects on the protein functions in addition to inhibiting its RNase activity and the *in vitro* assays used in the field (as well as in this study) do not seem to recapitulate the stringency of action of ISG20 in cells. It is important to note that although our results do not support a mechanism based on the direct degradation of target mRNAs by ISG20, they do not exclude that its exonuclease activity may be turned against a cellular RNA component, itself involved in translation. Rather, we favor this hypothesis in which ISG20 may attack ribosomal RNAs or small nucleolar RNAs that are required for ribosomal RNA methylation, possibly affecting the ribosomes’ behavior. Given that cellular RNAs continue to be translated, this implies that ISG20 may affect some, but not all ribosomes. We do not know whether this occurs here, however recent genome-wide studies have clearly highlighted that ribosomes are not only heterogeneous in composition, but also in function [30–33], so that it is possible that the activity of ISG20 is directed only against a specific subset of ribosomes.

The ability to discriminate RNAs according to the global definition of *self/non-self* origins we use here during translation is an interesting concept and STING has also been recently shown to inhibit the translation of viral, as well as of ectopically expressed mRNAs through a novel IFN-independent activity [34]. Notable differences exist however with the mechanism described here, as STING seems to act on the phase of translation initiation, thus sparing mRNAs that require no initiation factors (such as CrPV) [34], while on the contrary ISG20 targets also these mRNAs, strongly pointing in this case to a mechanism of regulation at a step other than translation initiation.

A previous study also suggested that ISG20 could interfere with viral translation in the absence of target RNA degradation [11]. In this study however, this inhibition was linked to the ability of ISG20 to induce IFN when expressed in MEF cell lines and more specifically the Interferon-Protein with Tetratricopeptide repeats 1 protein (IFIT1, [11]), a known translational modulator that impairs uncapped RNAs [25, 35, 36]. However, we have failed to obtain evidence of IFN and of IFIT1 upregulation in our experimental system and since IFIT1 cannot target the translation of mRNAs expressed from plasmid DNAs its involvement in the ISG20-mediated inhibition we describe here is unlikely. Furthermore, if ISG20 expression resulted in the direct upregulation of an IFN program, ISG20 would be expected to inhibit far more viruses than currently reported. It is therefore possible that this aspect of the biology of ISG20 can be observed solely under very particular conditions.

If translation initiation does not seem to be the main step affected by ISG20, our results highlight a tight correlation between the ability of the different ISG20 mutants to inhibit translation and their localization in P bodies, which is in line with the key role of these cytoplasmic structures in translational silencing and which would suggest RNA re-routing by ISG20. P bodies are involved in numerous aspects of RNA metabolism and although initially described as sites of RNA degradation [37], their functions have been further complexified by studies indicating their role in RNA storage as well as in RNA translational repression in the absence of RNA degradation [38–42]. Thus, the results we have obtained at this stage support the hypothesis that P bodies play an instrumental role in the action of ISG20. However, it remains possible that these structures form as a consequence of the translational block itself [43–45], so that additional work will be required to firmly establish the functional role of these structures during ISG20-mediated translation inhibition.

Our data indicate that the main determinant of susceptibility to ISG20 is the *self/non-self* nature of the genetic element from which the mRNAs originate and we hypothesize that the chromatin status of the transcribed elements may be a determining factor of this phenomenon. Specific co- or post-transcriptional modifications themselves linked to the chromatin status (as for example, N^6^-methyladenosine, m^6^A, reviewed in [46, 47]) are known to modulate different steps of mRNA biology and it is possible that a major difference between genetic elements embedded in the host chromatin and the others resides in the identity of marks deposited on the mRNA. Lack of such signals may expose the mRNA to ISG20 and to its translational regulation, providing an interesting level of regulation of foreign elements. An alternative and non-mutually exclusive hypothesis may be that the levels -and not the identity- of post-transcriptional modifications may be lower in elements that are either present in high number of copies or that are expressed at high levels, simply because the system is overrun, thus exposing the RNA to the action of ISG20. This hypothesis is also of interest and may be viewed as a basic threshold control mechanism of transcription/translation that is automatically turned on when genetic elements that replicate (or transcribe/translate) rapidly at high levels, as is the case during viral infection or following ectopic DNA transfection.

This hypothesis may certainly explain the different ISG20-susceptibility of RNA viruses with respect to cellular genes, because most RNA viruses replicate to exponential levels in cells in a very brief period of time, but it may also explain the ISG20-susceptibility of ectopically transfected DNAs/genes, as their chromatin status is distinct from the one of the host genome [19, 20, 48, 49] and their expression levels are generally much higher when compared to integrated cellular genes. In this respect, integration into the host genome or latent episomal maintenance may constitute a *self* mimicry defense strategy used by certain viruses, as we have shown here for EBV, to escape ISG20 surveillance. From a more general perspective, it will be of high interest to determine the extent of the translational control activity of ISG20 on viruses such as retroviruses, retroelements, as well as more broadly on different DNA viral families.

IFN responses rely on a complex network of more than 1.000 ISGs. Along with its multiple effects on the cell physiology, a major antiviral block of type I IFN responses takes place during translation initiation, by protein kinase R (PKR)-mediated inactivation of the eukaryotic initiation factor alpha (eIF2α, [50, 51]). Given that the effects of IFN on translation are not absolute and vary according to the cell type, the fact that ISG20 targets a step downstream translation initiation is of high interest, because it suggests the existence of a second layer of translational control in which ISG20 would target RNAs that have escaped the eIF2 α -block. By extension, this may suggest the existence of yet additional layer of translational control during IFN responses all of which concur in limiting viral replication.

Overall, our study reveals that ISG20 is an important modulator of antiviral responses *in vitro* and *in vivo* and points to a novel function through which this protein is able to distinguish *self* from *non-self* mRNAs at translation.

## Materials and methods

### Plasmids

N-terminal Flag-tagged human ISG20 mutants (Gene ID: 3669) were produced by overlapping PCR from an original pcDNA5/FRT-Flag-ISG20 plasmid obtained from Ju-Tao Guo (Baruch S. Blumberg Institute, Doylestown, Pennsylvania, USA) and cloned into pcDNA3 (Invitrogen), or pRetroX-Tight-Puro backbones by standard molecular biology techniques (Clontech). The latter is a murine leukemia virus (MLV)-based genome suitable for retroviral gene transduction and it allows for stable selection of recipient cells in which expression of the gene of interest is under the control of doxocycline of a modified *E*. *coli* Tet Repressor coded by a second MLV-based vector (pRetroX-Tet-On, rtTA-advanced, Clontech). Murine ISG20 (Gene ID: 57444) was obtained following the same cloning scheme. The remaining vectors were obtained as follows: pRRL-CMV-destabilized GFP (HIV-1-based lentiviral vector genome bearing an expression cassette for destabilized GFP, described in [52] and Clontech); Flag-TREX1 (in pcDNA3, this study), HIV-1 Gag (in [53], respectively), HA-Ubiquitin (gift of Pierre Jalinot, ENS-Lyon), VSV-tagged SAMHD1 (in pRRL-CMV, this study); HA-destabilized TNRC6A was engineered by standard molecular biology techniques from the double-tag pFRT/TO/FLAG/HA-Dest-TNRC6A (gift from Thomas Tuschl, Addgene, #19883, [54]). An N-terminal HA tagged IFIT1 (Gene ID: 3434) was cloned by standard molecular biology techniques into pcDNA3.1 (Invitrogen). The following bicistronic vectors were used to examine the role of different IRESes and were described in [55] and [22]: Bi-Glo-Fluc-cMyc -RLuc; Bi-Glo-Fluc-EMCV-RLuc and Bi-Glo-Fluc-PV-RLuc. The following constructs code for a Renilla Luciferase gene under the control of the beta-globin promoter and contain or lack an intron: pGlobin-intron-RLuc and pGlobin-RLuc (described in [22], gift from Fabrice Mure, CIRI, Lyon, France). The following vectors have been used for in vitro production of RNA and they allow upon linearization the T7-based synthesis of a polyadenylated mRNA that is then 5’ capped: pHCV-Rluc, pCrPV-Rluc, pcMyc-RLuc. The pVSV-Rluc was constructed by standard molecular biology techniques by placing the region 1–63 upstream of the N gene of VSV (EF197793) in front of the Renilla Luciferase. Upon synthesis, RNAs are DNase treated, precipitated and used for cell transfection.

### Antibodies

Antibodies directed against the following proteins were as follows: Tubulin (T0198), Actin (A5441), Flag (F7425 and F3165), GFP (G1544), HA (H3663), VSV-G (C7706), TNRC6A (HAP015305) from Sigma; mSin3A (ab129087); donkey anti-rabbit IgG (Alexa Fluor 594 conjugate, A-21207) and donkey anti-mouse IgG (Alexa Fluor 488 conjugate, A-21202) from Invitrogen; CREB (4820) and Akt (9272) from Cell Technology; DDX6 (BET1300-460A) from Ozyme; ISG20 (22097-1-AP, in our hands this antibody is highly unreliable on human ISG20 but is more efficient on murine ISG20) from Proteintech; sc35 (NB100-1774) from Novus Biologicals; anti BMRF1 (MAB8186, Merck Millipore); anti BMLF1, anti BRLF1 and BZLF1 were described in [56, 57]; anti-IFIT1 (PA3-848, Life Technologies). The anti-Gag/p24 antibody (clone 183-H5C) was obtained from the AIDS Reagents Program of the NIH.

### Cells

Human embryonic kidney 293T (HEK293T) and human cervix epithelial HeLa cells (HeLa) were maintained in complete DMEM media supplemented with 10% FCS, while human peripheral blood T lymphocyte Jurkat (Jurkat), human peripheral blood monocytic THP-1 cells (THP-1) and U937 were maintained in complete RPMI1640 medium with 10% FCS. Cells were obtained from the ATCC (CRL-3216; CCL-2; TIB-152; TIB-202, CRL-1593.2 respectively). HEK293T-EBV [58], Hone [21] and Raji cells were obtained from the laboratory of Oncogenic Herpesviruses of Henri Gruffat (CIRI, Lyon) and were maintained in complete DMEM or RPMI1640, respectively. EBV reactivation was induced in Hone cells upon incubation with 12-O-tetradecanoylphorbol 13-acetate (*TPA*) and butyric acid (Ba) provided at 50 ng/ml and 3 mM, respectively (SIGMA). When indicated the proteasome inhibitor MG132 was added at a final concentration of 10 μg/ml (Sigma). HL116 cells, derivative of HT1080 cells that bear an integrated *luciferase* under the control of the interferon alpha-inducible protein 6 promoter (6–16:Luc) were obtained from Gilles Uze (University of Montpellier, Montpellier, France [59]) and were maintained in complete DMEM.

Primary human blood monocytes were obtained by negative depletion as described in [52] (monocyte isolation kit II, catalogue n° 130-091-153, Miltenyi) prior to their differentiation in macrophages or dendritic cells upon incubation for 4 to 6 days in M-CSF or GM-CSF and IL4 (at 100 ng/mL, 01-A0220-0050, PCYT-221 and PCYT-211, Eurobio). When indicated, cells were incubated for further 24 hours with 1.000 U/mL of human IFNα (catalogue n° 11100–1, Tebu Bio), or in the case of murine BMDM with 1.000 U/mL of murine IFNβ (50708-M02H, Sino Biological Inc.).

Primary mouse bone marrow derived macrophages (BMDMs) were prepared from *isg20-/-* and *isg20+/+* mice, as described before [60]. Briefly, bone marrow cells were isolated from mice femurs and tibias, washed with PBS and filtered through a 70 μm cell strainer to remove cell clumps. The single-cell suspension was then cultured in RPMI1640 medium containing 10% FBS and 20ng/ml of M-CSF (416-ML, R&D Systems) for 7 days with daily media replenishment. Differentiation was monitored by flow cytometry analysis of CD11b+F4/80+ cells (BD) and reached proportion higher than 90%.

### Stable cells

Pools of stable cell lines allowing for doxycycline (dox)-inducible ISG20 expression were generated upon MLV-based retroviral gene transduction based on the pRetroX-Tight system of Clontech. Briefly, the corresponding MLV vectors were produced by transient DNA transfection of HEK293T cells with plasmids coding the structural MLV protein Gag-Prol-Pol, the pantropic Vesicular Stomatitis Virus G protein (VSVg), pRetroX-Tight-Puro and pRetroX-Tet-On vectors that code for a miniviral genome bearing expression cassettes for a resistance gene (Puromycin and Neomycin, respectively) and either ISG20, or the tetO transcriptional rtTA activator (ratios 8:4:4:4, respectively, for a total of 20 μg of transfected DNA for a 10-cm dish). Virion particles released in the supernatant of transfected cells were filtered, titered by exogenous RT activity [60] and used to challenge the indicated target cells. Pools of transduced cells were obtained upon selection with Puromycin and G418 (P9620 and G418-RO, Sigma). ISG20 expression was induced by incubation with doxycycline (631311, Clontech).

### Transient DNA or RNA transfections

For mRNA transfection, 10^5^ HEK293T cells were electroporated in quadruplicates with 200 ng of *in vitro* transcribed capped and polyA mRNA in a total volume of 10 μl (settings of 1150V, 20ms, 2 pulses, Neon electroporator, Invitrogen). Cells were then transferred into 500 μl pure RPMI 1640 medium and cultured for 1 hour prior to cell lysis and luciferase analysis (see below). mRNAs were generated by an in-house *in vitro* transcription reaction and then capped. Briefly, 1 μg of linearized DNA was incubated for 2 hours at 37°C with 20U of T7 RNA polymerase, 40 U of RNAsin (both from Promega) and 1.6 mM for each ribonucleotide triphosphate, 3 mM DTT, 40mM Tris–HCl (pH 7.9), 6 mM MgCl_2_, 2 mM spermidine, and 10 mM NaCl. To induce mRNA cap addition, the rGTP concentration was reduced to 0.32 mM, and 1.28 mM of m^7^GpppG cap analog (New England Biolabs) was added. RNAs were then precipitated and finally resuspended in RNAse-free water. DNA transfections were carried out generally by calcium/phosphate transfection, or Lipofectamine 2000 for confocal microscopy (Invitrogen). Generally, for transient DNA transfections a ratio of 1:1 between reporters and ISG20 was used (from 1:1 to 1:8 in the dose curve presented in S4 Fig) not surpassing an overall amount of 3 μg of ISG20 per well of 6 well plate to avoid cytotoxic effects.

### Luciferase assays

Cells were analyzed in triplicates and lysed between eighteen and twenty-four hours after DNA, or 1 hour after RNA transfection. One fifth of the cell lysates was used for *Firefly* or *Renilla* luciferase assays (Luciferase Assay System or *Renilla* Luciferase Assay System, Promega; Mithras LB 940 Multimode Microplate Reader, Berthold), while the remaining fractions were used for RNA extraction and quantitative real-time PCR assay. The luciferase activities are normalized for the amount of RNA present in each sample.

### Metabolic labelling

Cells were starved in 1 ml/well of Met-/Cys-free DMEM medium (21013024, Gibco) for 1 hour and then medium was replaced with 500 μl of the same medium containing 20 μCi ^35^S Met/Cys (Amersham) for a pulse of 1 hour. Labeled cells were then lysed thoroughly with RIPA buffer (150 mM NaCl, 1% NP40, 0.5% deoxycholate, 0.1% SDS, 50 mM TrisCl pH 8.0) in the presence of protease inhibitors (Roche), by vigorous pipetting and 3 cycles of freeze-thawing. Lysates were centrifuged at 12.000 g for 10 minutes to remove insoluble material and then either directly analyzed by SDS-PAGE gels or incubated with 20 μl anti-M2-Flag beads for 4 hours to overnight at 4°C. After extensive washing, material was separated by SDS-PAGE gel and upon gel fixation in methanol and drying, radioactivity was quantified by phosphor imager (Typhoon 8600 system, GE healthcare).

### Confocal microscopy

Stable or transfected cells were directly grown on coverslips for at least twenty-four hours prior to fixation in cold methanol for 10 min at -20°C (HeLa or HEK293T cells, respectively). When indicated, a prior permeabilization step was carried out to evidence the strong association of ISG20 to nuclear bodies. In this case, cells were transfected in duplicate and while one aliquot was immediately fixed, the other was first washed in cold wash buffer (PBS, 0.2% Tween 20) and further permeabilized in cold PSB containing 0.5% Triton-X100 for 5 min on ice. Cells were then fixed and incubated with the indicated antibodies (see relevant section above). DAPI Fluoromount G mounting medium was used (0100, Southern biotech). Images were acquired using a spectral Zeiss LSM710 or LSM800 confocal microscopes and analyzed using the Fiji software. For P bodies analyses, cells were fixed first in 4% paraformaldehyde, then in 70% methanol (10 min each), followed by permeabilization in cold PSB containing 0.5% Triton-X100 for 5 min on ice.

### *In vitro* exonuclease activity assays

Cells either stably or transiently expressing ISG20 were lysed in pre-cooled buffer SD-150 (150 mM NaCl, 50 mM Tris pH7.4, 0.5% Triton X100, 0.5 mM MnCl_2_ and 1× Roche Protease inhibitor Cocktail), sonicated and the soluble supernatants were incubated with pre-washed anti-FLAG antibody-conjugated agarose beads (A2220, Sigma, 20μl per 10-cm plate dish) for 4 hours at 4°C with constant nutation. Beads were then extensively washed in the same buffer and either analyzed by WB or incubated with 0.5–1 μg of exogenously added nucleic acids for 60 minutes at 37°C in a total volume of 30 μL of SD-150 buffer, prior to agarose gel migration and densitometric quantification by Multi Gauge V3.0 software (Fujifilm). Sequences of the complementary DNA/RNA oligonucleotides are as follows: DNA oligonucleotides D20 and D15 were respectively: ACATGTACAGGATGCATTTG and TACAGGATGCATTTG. RNA oligonucleotides R20U, R20D and R20D were respectively: CAAAUGCAUCCUGUACAUGU, ACAUGUACAGGAUGCAUUUG, UACAGGAUGCAUUUG. Hybrids were obtained by mixing the desired oligonucleotides at a concentration of 1 μg in SD-150 buffer, prior to denaturation at 95°C and annealing at 50°C for 5 minutes each. Poly I:C and yeast tRNAs were purchased from Invivogen and Sigma, respectively. ssRNA was a Luciferase based mRNA generated by in vitro transcription of approximatively 1700 nucleotides. ssDNA and dsDNA were respectively a long chemically-synthesized oligonucleotide available in the laboratory for other purposes (Eurogentec, CGCGGAA TTCACCATGGATTACAAGGATGACGACGATAAGGGTGGTGGTTCAGCCCCACTGGATGCCGCCCTCCAC) and double-stranded pcDNA3 plasmid (Invitrogen) linearized with PstI.

### IFN quantification

The levels of type I IFNs secreted in the supernatant of cells expressing ISG20 was determined according to a well established procedure using HL116 reporter cells [59]. Briefly, ISG20-stable U937 cells were stimulated for 24 hours with doxycyclin to induce ISG20 expression, then supernatant was harvested, filtered and added to HL116 cells in serial dilution (2.10^4^ cells in 96-well plate). Twenty hours afterwards, cells were lysed and the extent of luciferase accumulation was measured (see above, Luciferase assays section). HL116 cells stimulated with serial dilutions of IFNα were used in parallel to ensure the linearity of the assay. The limit of detection of this assay is in our hands of 30 U/mL.

### Quantitative PCRs and RT-qPCRs

RNAs were extracted by the Trizol Reagent (15596018, Ambion), or by NucleoSpin RNA (740955, Macherey-Nagel) according to the manufacturers’ instructions. When indicated, cytoplasmic and nuclear RNAs were separated and purified, as previously described (Ricci EP, PMID: 19528074). The extracted RNA was treated with RQ1 DNase to remove contaminant DNA (M6101, Promega) and then reverse-transcribed by qScript cDNA SuperMix using the manufacturer’s instructions and either oligo-dT or random primers (Quanta BioSciences; the enzyme was instead omitted in the no-RT controls). When indicated, the amount of transfected DNA was quantified after the addition of RQ1-DNase to the cell culture media to remove extracellular DNA (twice for two hours at 37°C at 20 U/ mL). After extensive cell washing, cells were lysed with the following solution (50 mM KCl, 10 mM Tris HCl pH 8.3, 2.5 mM MgCl_2,_ 0.5% NP-40, 0.5% Tween 20) in the presence of 10 μg/ml of Proteinase K (P2308, Sigma) for one hour at 60°C. DNA was then purified through phenol/chloroform extraction and DNA precipitation. qPCRs were performed on a StepOne Plus Real-time PCR system (Applied Biosystems) using the FastStart Universal SYBR Green Master mix (Roche). Primers were as follows (forward-F and reverse-R from 5’ to 3’): gfp-amplicon-(a) (F: TGAGCAAGGGCGAGGAGCTGTTCACC; R: CTTCAGGGTCAGCTTGCCTAG GTG); gfp-amplicon-(b) (F: GAACGGCATCAAGGTGAACT; R: TGCTCAGGTAGTGGTTGTCG); Renilla (F: AGGTGAAGTTCGTCGTCCAACATTATC; R: GAAACTTCTTGGCACCTTCAACAATAGC); hprt1 (F: TGACCTTGATTTATTTTGCATACC; R: CGAGCAAGACGTTCAGTCCT); actin (F: TTTTCACGGTTGGCCTTAGG; R: AAGATCTGGCACCACACCTTCT); U1 (F: CTTACCTGGCAGGGGAGATACC; R: GCAGTCGAGTTTCCCACATTTGG); U2 (F: GCTTCTCGGCCTTTTGGCTAAG; R: CAATACCAGGTCGATGCGTGG); U3 (F: CGTGTAGAGCACCGAAAACCAC; R: CTCCCCAATACGGAGAGAAGAACG); U6 (F: CGCTTCGGCAGCACATATAC; R: GGAACGCTTCACGAATTTGCG).

### Fractionations

Cytoplasmic and nuclear fractions were obtained from transfected HEK293T cells. Briefly, 0.5–1.10^6^ cells were collected and resuspended in 0.2 ml of cold RNLa buffer (10 mM of Tris HCl pH 8.1, 10 mM NaCl, 3 mM MgCl_2_, 0.5% NP-40, 1 mM DTT and 100 U/ml of RNasin Plus-Promega) for 5 minutes on ice, then centrifugated at 1.200 g for 5 minutes at 4°C. The supernatant (cytoplasmic fraction) was transferred to a new tube, while the pellet (nuclear fraction) was washed twice with 0.2 ml of cold RNLa buffer to eliminate residual cytoplasm and discarded. The pellet was then resuspended with 0.2 ml of cold RNLa buffer, sonicated on ice for 10 seconds. An aliquot of each fraction was stored for WB analysis, while the rest was used for RNA extraction (Trizol, Thermo Fisher Scientific). Results are presented on normalized cytoplasmic and nuclear fractions. Ribosomal fractions were purified as described [61]. Briefly, cells at less than 80% confluency were washed in cold PBS, gently pelleted and lysed for 10 minutes in hypothonic buffer (10 mM KCl, 0.5 mM MgCl_2_, 10 mM Tris-HCl pH 7.4). Nuclei were pelleted by centrifugation at 800 g for 10 minutes and the supernatant was then transferred centrifuged again to remove mitochondria (12.000 g for 10 minutes). Supernatant was then harvested, adjusted to 0.5 M of KCl and placed onto a sucrose cushion (34.2% w/v) prior to ultracentrifugation at 75 000 rpm on a TL100 ultracentrifuge (for two hours at 4°C). After ultracentrifugation, the supernatant constitutes the cytoplasmic fraction remaining after migration of the ribosomes at the bottom of the tube.

### Viral infections

The replication-competent VSV-GFP (Indiana serotype, [18]) contains an additional viral transcriptional unit coding GFP between M and G and was kindly provided by Joanna Brunel and Denis Gerlier (CIRI, ENS-Lyon). Infections of ISG20 expressing cells were carried out generally MOIs comprised between 0.001 and 0.01, twenty-four hours after dox-induction of ISG20. The percentage of GFP-positive cells was determined at different time points post-infection by fluorescence-activated cell sorting (FACS).

### Generation of ISG20-knockout mice and animal infections

ISG20-knockout mice were generated by clustered regularly interspaced short palindromic repeats (CRISPR)-associated systems (CRISPR/Cas9). Briefly, a single ISG20 sgRNA was designed to target the mouse *isg20* ORF (5’-GATCACTAATACGACTCACTATAGGTGGGCCTCAAAGGGTGAGGTTTTAGAGCTAGAAAT-3’). *In vitro* Transcription T7 Kit (6140, TaKaRa) was used for transcription of both sgRNA and Cas9, the RNA products were purified and injected into C57BL/6 mice zygotes [60]. Screening of knockout mice was carried out by PCR on genomic DNA from F0 and F1 mice followed by PCR products cloning and sequencing (TA cloning kit, TaKaRa: F: TTTCTGAGGGTCGCCAA and R, TGTACTTGTCATACAGGACT)

For *in vivo* VSV replication studies, age- and sex-matched groups of littermate mice were intraperitoneally infected with 10^8^ plaque-forming units (pfu) of WT-VSV (Indiana serotype, but in this case lacking GFP, [60]). Mice were then either sacrificed twelve hours after infection for tissue analysis and in the case of lung sections for staining with hematoxylin and eosin that marks preferentially inflammatory cells or kept for prolonged periods of time for survival studies.

PEMs were harvested by peritoneal washes from mice treated 3 days before via an intraperitoneal injection of 3 ml of 4% sterile thioglycollate (Sigma T9032). The cell suspension was filtered through a 70-μm cell strainer (Falcon 352350) to remove cell clumps and plated at 0.5 million per well in 24-well plate in complete 1640 medium to force cell adhesion to plastics and then cells were extensively washed to remove non-adherent cells. VSV infections were performed the next day at an MOI of 0.5 prior to flow cytometry analysis twenty-four hours later.

### Ethics statement

Primary blood cells were obtained from the blood of healthy donors (EFS-Lyon) as discarded “leukopacks”. These were obtained anonymously so that gender, race, age of donors, inclusion of women, minorities or children cannot be determined. This research is exempt from approval and was therefore not submitted to Institutional Review Board approval, although written informed consent was obtained from blood donors to allow use of their cells for research purposes.

All animal experiments described in this study were performed according to the regulations of the Association for Assessment and Accreditation of Laboratory Animal Care in Shanghai and the Animal Investigation Committee of East China Normal University (document approval n° M20130105). C57BL/6 wild-type mice were purchased from the Shanghai Laboratory Animal Company. Mice were housed in a temperature and humidity-controlled room (21°C +/-1°C and 55% +/-10%, respectively) with a 12-hour light/12-hour dark cycle. Throughout the protocol, animals were weighed and observed twice daily for clinical signs of infection. Animals that reached the end of the experiment or lost more than 20% of initial body weight were sacrificed.

## Supporting information

S1 Fig Expression levels and cytotoxicity of ISG20. A) The levels of ISG20 mRNAs were quantified by RT-qPCR in the different cell types indicated. Monocyte-derived macrophages and dendritic cells (MDM and DCs, respectively) were generated upon incubation of primary blood monocytes with M-CSF or GM-CSF and IL4, respectively for 4 to 6 days as described in [60]. When indicated, cells were incubated for twenty-four hours with 1.000 U/mL of interferon α, prior to cell lysis. The expression levels of ISG20 obtained in dox-inducible stable cell lines generated upon retroviral mediated gene transduction are also shown (dox concentrations of 0, 1 and 10 μg/ml). B) The cytotoxicity of ISG20 was evaluated in stably transduced cell lines in which ISG20 induction is dox-sensitive. Cells were counted daily upon induction of ISG20 with dox concentrations of 0,1 and 10 μg/ml. The graph presents data obtained from three to four independent experiments, while the WB panels present typical results obtained.
(TIF)

S2 Fig The antiviral activity of ISG20 is observed at different viral inputs. HeLa cells expressing WT-ISG20 were induced with 1 μg/ml dox, as in the legend to Fig 1 and then infected with the indicated viral inputs of VSV-GFP. The extent of viral spread was measured by flow cytometry over time. The panel presents one representative replication experiment.
(TIF)

S3 Fig Representative flow cytometry panels and Mean Fluorescence Intensity (MFI) analyses. The figure depicts representative FACS panels obtained during VSV infection. The percentage of GFP-positive cells is displayed in each panel. The graph presents variations of the MFI in GFP-positive populations in the different conditions corresponding to the latest two points of the replication curves of Fig 1A. *, p≤0.05 according to a Student t test comparing control and ISG20 conditions.
(TIF)

S4 Fig Exonuclease activities and specificities of ISG20 *in vitro* and in cells. A and B) HEK293T cells transiently transfected in 10 cm plates with ISG20 coding DNA were lysed and ISG20 immunoprecipitated via anti-flag antibodies conjugated to agarose beads. After washing, beads bound material was incubated with the indicated nucleic acids (1 μg), prior to loading on agarose gels and densitometry quantification. Nucleic acids were as follows: ssRNA, *in vitro* transcribed single-stranded RNA, length of approximatively 1700 nucleotides; ssDNA, single-stranded DNA, oligonucleotide of 76 nucleotides; dsDNA, double stranded pcDNA3 plasmid (Invitrogen) linearized with PstI; poly I:C and yeast tRNAs, self-explicatory. In B, the indicated RNA and DNA forms were generated as described in the Methods section. C) HEK293T cells transiently transfected as above were directly lysed and the amount of small nuclear RNAs (U1, U2, U3 and U6) or of total RNA was evaluated by RT-qPCR and agarose migration, respectively. D) As in C but cells were also transfected with 200 ng of *in vitro* transcribed mRNA containing the 5’ UTR of the hepatitis C virus (HCV) and bearing the Firefly luciferase. Upon cell lysis the amount of transfected HCV-Luc mRNA was determined by RT-qPCR. A schematic representation of the RNA target and the position of the PCR amplicon is provided. The graphs present Means and SEM of independent experiments (n = 2 to 3 depending on target for B; n = 3 for C; n = 4 for D). The panels present typical results obtained. *, p≤ 0.05, following a Student t test.
(TIF)

S5 Fig The effects of ISG20 on translation are not due to higher proteasomal targeting. HEK293T cells were transiently transfected with a plasmid coding for GFP reporter and incubated with 10 μg/ml of MG132 (Sigma) overnight, prior to cell lysis and WB analysis. The panels present typical results obtained.
(TIF)

S6 Fig Effects of ISG20 on RNA integrity and translation of ectopic substrates. A) RNA obtained from HEK293T cells transiently transfected as in Fig 2B was reverse transcribed with either oligo-dT or random hexamers and then amplified with the indicated primers placed at different locations in the target GFP mRNA (the amplicon referred to as b is the one routinely used in the remaining figures). The graph presents results of 3 independent experiments. B) HEK293T cells transiently transfected with ISG20 and GFP coding plasmids (as in Fig 2B) were analyzed by WB twenty-four hours afterwards. To better appreciate the linearity and magnitude of the defect in GFP accumulation by WB, the control sample was serially diluted. C) As above, but cells were analyzed by flow cytometry to appreciate the decrease in the percentage of GFP-positive cells (displayed in the panels), as well as their MFI. D) HEK293T cells were transiently transfected with a fixed dose of GFP reporter and increasing concentrations of ISG20 followed by cell analysis by WB (from 1:1 to 1:8). E) HEK293T cells were transfected with DNAs coding for indicated proteins using either Calcium phosphate or lipofectamine-based DNA transfection, prior to WB analysis.
(TIF)

S7 Fig Characterization of ISG20 mutants. A) HeLa cells expressing the different ISG20 mutants were challenged with an MOI of 0.01 of VSV-GFP and the extent of viral infection was measured at a single time point, fifteen hours afterwards by flow cytometry. B) HEK293T cells were transfected with DNAs coding the different ISG20 mutants along with a GFP reporter. The amount of GFP reporter was then determined by densitometry after WB. C) The different ISG20 mutants were immunoprecipitated from transfected HEK293T cells and incubated with a single-stranded RNA oligo (R20). Upon migration on an agarose gel, the amount of intact RNAs were measured by densitometry. D) Correlative analysis between the RNAse and antiviral properties of individual ISG20 mutants. The graph present Means and SEM of four individual experiments (two for panel B). *, p≤ 0.05 following a Student t test. E) Spatial positioning of the indicated mutations on the crystal structure of ISG20.
(TIF)

S8 Fig ISG20 does not affect the production of infectious virion particles of EBV. Hone cells containing a latent EBV genome bearing GFP were transfected with ISG20 and EBV reactivated from latency upon TPA/BA treatment as in the legend to Fig 2D. To determine whether ISG20 could affect the translation of the plethora of virion products required for the production of infectious virion particles, supernatants were syringe-filtered and virion infectivity was measured after challenge of target Raji cells and flow cytometry analysis three days later.
(TIF)

S9 Fig ISG20 does not influence nucleocytoplasmic RNA transport and is not detectably associated to ribosomes. A) HEK293T cells were cotransfected with ISG20 along with two constructs coding for a Firefly luciferase reporter expressed from a globin promoter and containing or not an intron, prior to cell lysis and luciferase measurement. B) HEK293T cells were transiently transfected with ISG20 and GFP-coding plasmids and subsequently lysed to obtain nuclear and cytoplasmic fractions. Upon normalization of the two fractions by volume, samples were analyzed by WB and RT-qPCR (GFP). The distribution of two small nuclear RNAs known to be enriched in the nucleus (U1 and U3 by RT-qPCR) was also included as control for fractionation. C) HEK293T cells transfected with ISG20 were lysed, the ribosomal fraction purified, followed by WB analysis. The graphs present data obtained from two (Luc) and five (PCRs) independent experiments and panels present typical results. *, p≤ 0.05, following a Student t test.
(TIF)

S10 Fig The catalytically inactive M1-ISG20 mutant is devoid of translation inhibition activity. HEK293T cells were transfected with the indicated reporters along with WT or M1-ISG20 mutant prior to cell lysis and luciferase assay measurement. The graph presents data obtained from two and four independent experiments. Glo, globin; PV, polyomavirus; Myc, c-Myc. The nomenclature 1^st^ and 2^nd^ refers to the position of the cistron in bicistronic vectors.
(TIF)

S11 Fig ISG20 and IFIT1 inhibit VSV infection. HEK293T cells transfected with plasmids coding the above-mentioned proteins were challenged twenty-four hours afterwards with VSV-GFP at an MOI of 0.01 prior to flow cytometry analyses twenty four hours later. The graph presents results obtained with three independent experiments. *, p≤ 0.05, following a Student t test between control cells and the indicated condition.
(TIF)

S12 Fig ISG20 displays a weak association to nuclear bodies and does not modify the extent of P bodies formation in the cell. A) HEK293T cells were transiently transfected in duplicate with plasmids coding ISG20 (routine transfection rates ≥80%). Twenty-four hours afterwards, one aliquot was immediately fixed while the second was first permeabilized with detergent prior to fixation. Both were then similarly processed and analyzed by confocal microscopy using antibodies specific for ISG20 (Flag), as well as for the nuclear speckles marker SC35. This procedure is commonly used to study nuclear bodies and associated proteins that resist detergent extraction prior to fixation. Representative pictures and relative distributions are show here (>80 cells scored per condition). B) Control cells were analyzed by confocal microscopy with the endogenous P bodies markers TNRC6A and DDX6 (ISG20-expressing cells and a portion of the overlay of control cells depicted here are presented in Fig 5A).
(TIF)

S13 Fig Alignment of murine and human ISG20s. Amino acids alignment of human and murine ISG20s. The black bar presents the CRISPR/Cas9 target sequence used. Shaded boxes indicate the position of the exonuclease domains I to III.
(TIF)
