# Correction: The interferon stimulated gene 20 protein (ISG20) is an innate defense antiviral factor that discriminates *self* versus *non-self* translation

**DOI:** 10.1371/journal.ppat.1011176

**Published:** 2023-02-16

**Authors:** Nannan Wu, Xuan-Nhi Nguyen, Li Wang, Romain Appourchaux, Chengfei Zhang, Baptiste Panthu, Henri Gruffat, Chloé Journo, Sandrine Alais, Juliang Qin, Na Zhang, Kevin Tartour, Frédéric Catez, Renaud Mahieux, Theophile Ohlmann, Mingyao Liu, Bing Du, Andrea Cimarelli

In Fig 7B, the x axis should be labeled Hours post-infection. Please see the corrected figure below.

**Fig 7:**
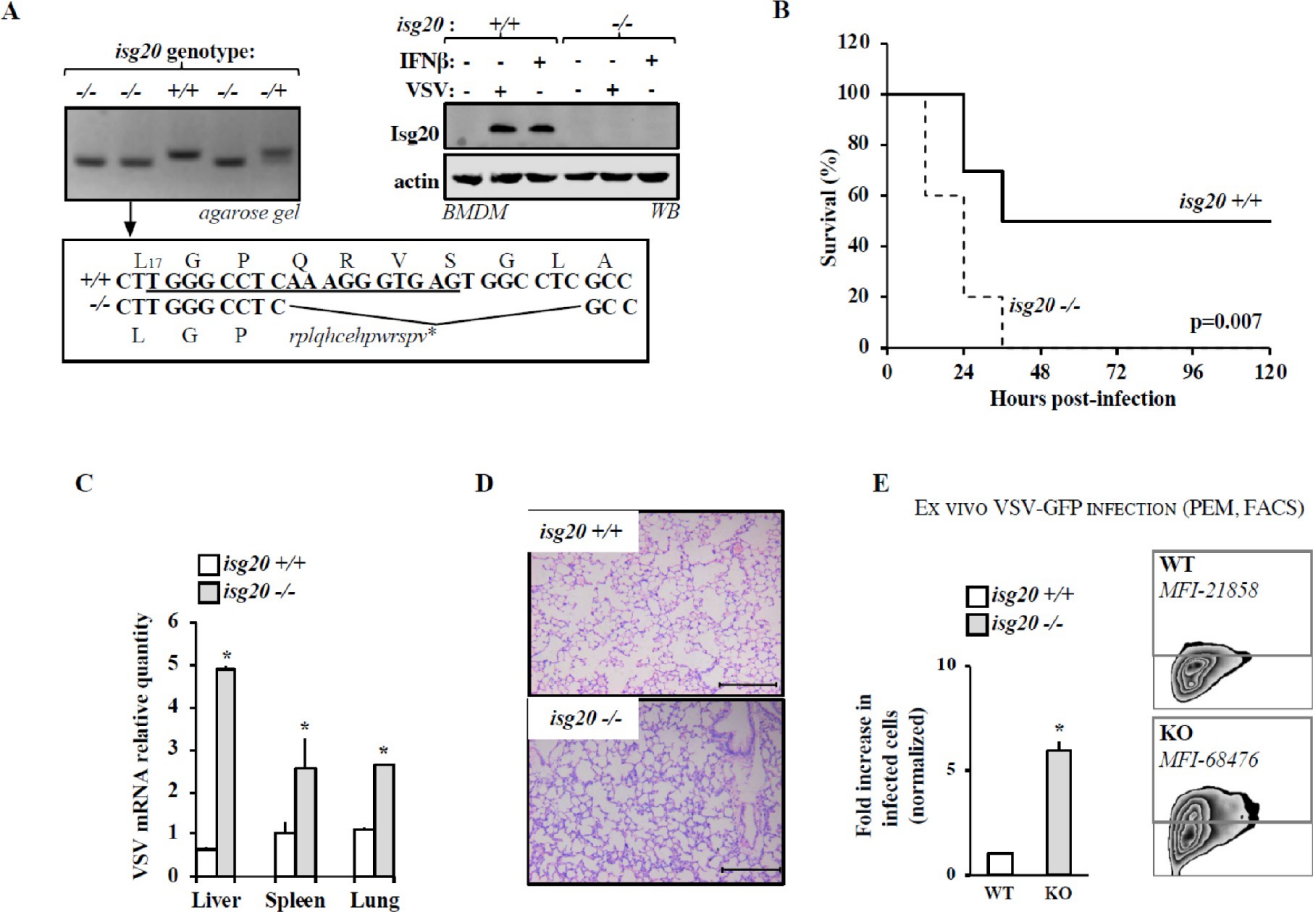
*Isg20* -/- mice exhibit higher susceptibility to VSV infection. A) CRISPR/Cas9 was used to generate a functional ISG20 knockout mouse. The target sequence is underlined and the resulting indel mutation and downstream open reading frame, as well as corresponding PCR in founders, are shown. To detect ISG20 protein, bone marrow-derived macrophages were stimulated either with IFN β or with VSV for eight to twelve hours prior to WB analysis. B) *isg20*-/- and +/+ age- and sex-matched littermates were intraperitoneally infected with VSV. The graph presents survival rates of each group. C) The extent of VSV replication in the indicated tissues was determined by RT-qPCR. D) Representative lung sections are shown upon hematoxylin and eosin staining that preferentially label inflammatory cells. E) Peritoneal macrophages (PEMs) were prepared from *isg20* -/- or control mice and then challenged *ex vivo* with VSV-GFP (MOI 0.5) prior to flow cytometry analysis twenty-four hours later. A representative zebra plot is shown with respective MFI of GFP-positive cells. The graphs present Means and SEM of three independent experiments (20 animals in two independent experiments in survival experiments).
