# Supplementary figures and images for: The interferon stimulated gene 20 protein (ISG20) is an innate defense antiviral factor that discriminates self versus non-self translation
Source: PLoS Pathog. 2019 Oct 10;15(10):e1008093. doi: 10.1371/journal.ppat.1008093 (PMC6805002; doi:10.1371/journal.ppat.1008093)

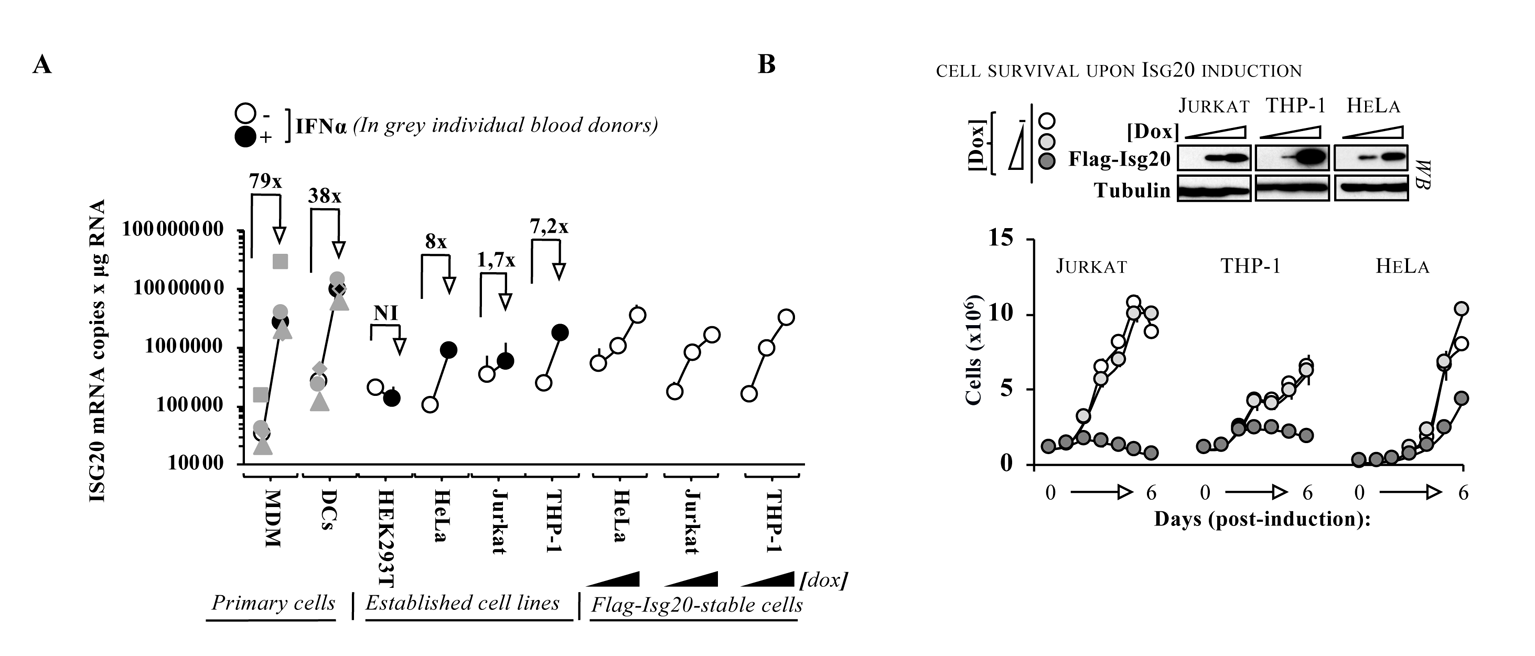

Supplement: S1 Fig — A) The levels of ISG20 mRNAs were quantified by RT-qPCR in the different cell types indicated. Monocyte-derived macrophages and dendritic cells (MDM and DCs, respectively) were generated upon incubation of primary blood monocytes with M-CSF or GM-CSF and IL4, respectively for 4 to 6 days as described in [60]. When indicated, cells were incubated for twenty-four hours with 1.000 U/mL of interferon α, prior to cell lysis. The expression levels of ISG20 obtained in dox-inducible stable cell lines generated upon retroviral mediated gene transduction are also shown (dox concentrations of 0, 1 and 10 μg/ml). B) The cytotoxicity of ISG20 was evaluated in stably transduced cell lines in which ISG20 induction is dox-sensitive. Cells were counted daily upon induction of ISG20 with dox concentrations of 0,1 and 10 μg/ml. The graph presents data obtained from three to four independent experiments, while the WB panels present typical results obtained. (TIF) [file ppat.1008093.s001.tif]

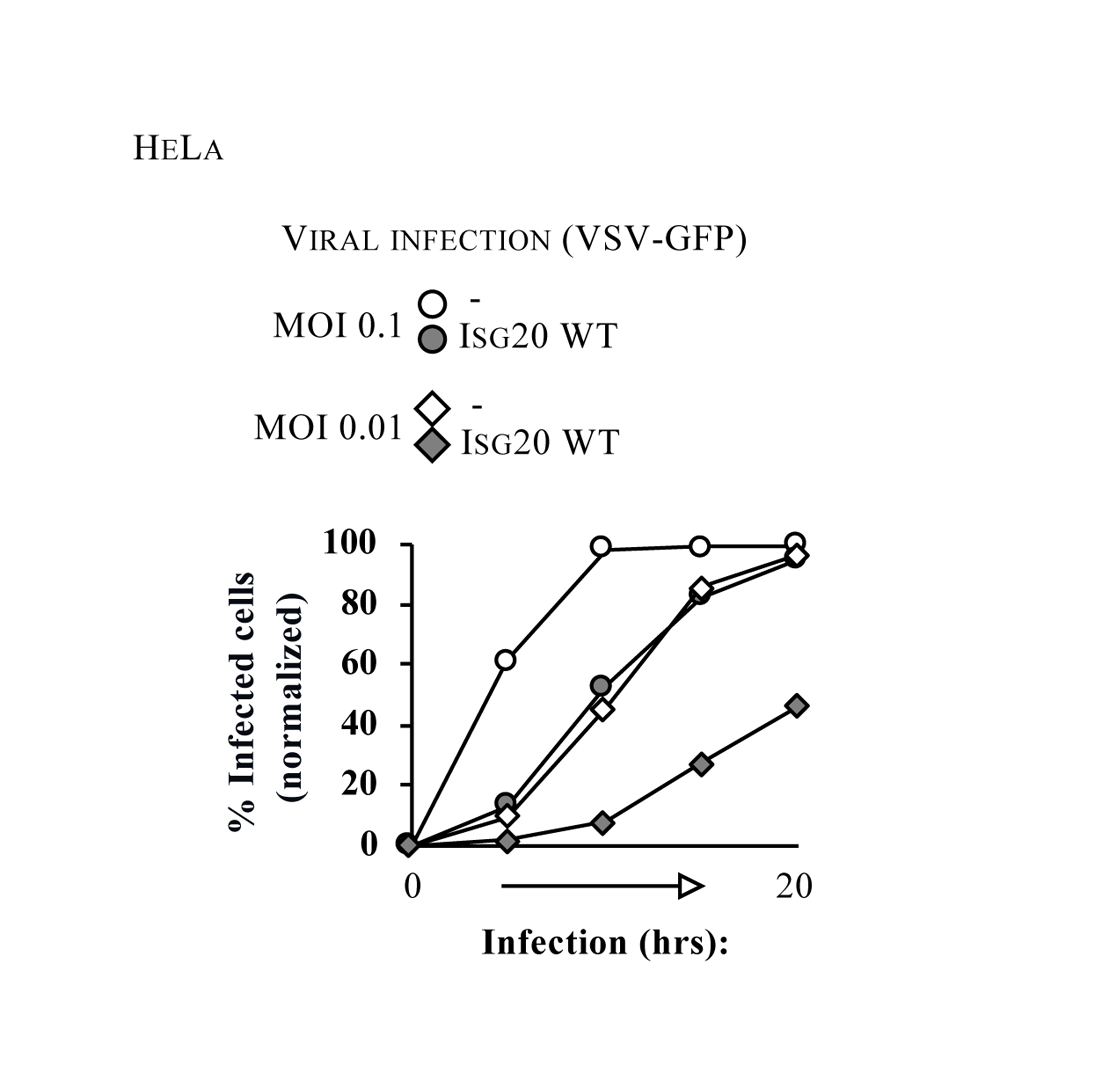

Supplement: S2 Fig — HeLa cells expressing WT-ISG20 were induced with 1 μg/ml dox, as in the legend to Fig 1 and then infected with the indicated viral inputs of VSV-GFP. The extent of viral spread was measured by flow cytometry over time. The panel presents one representative replication experiment. (TIF) [file ppat.1008093.s002.tif]

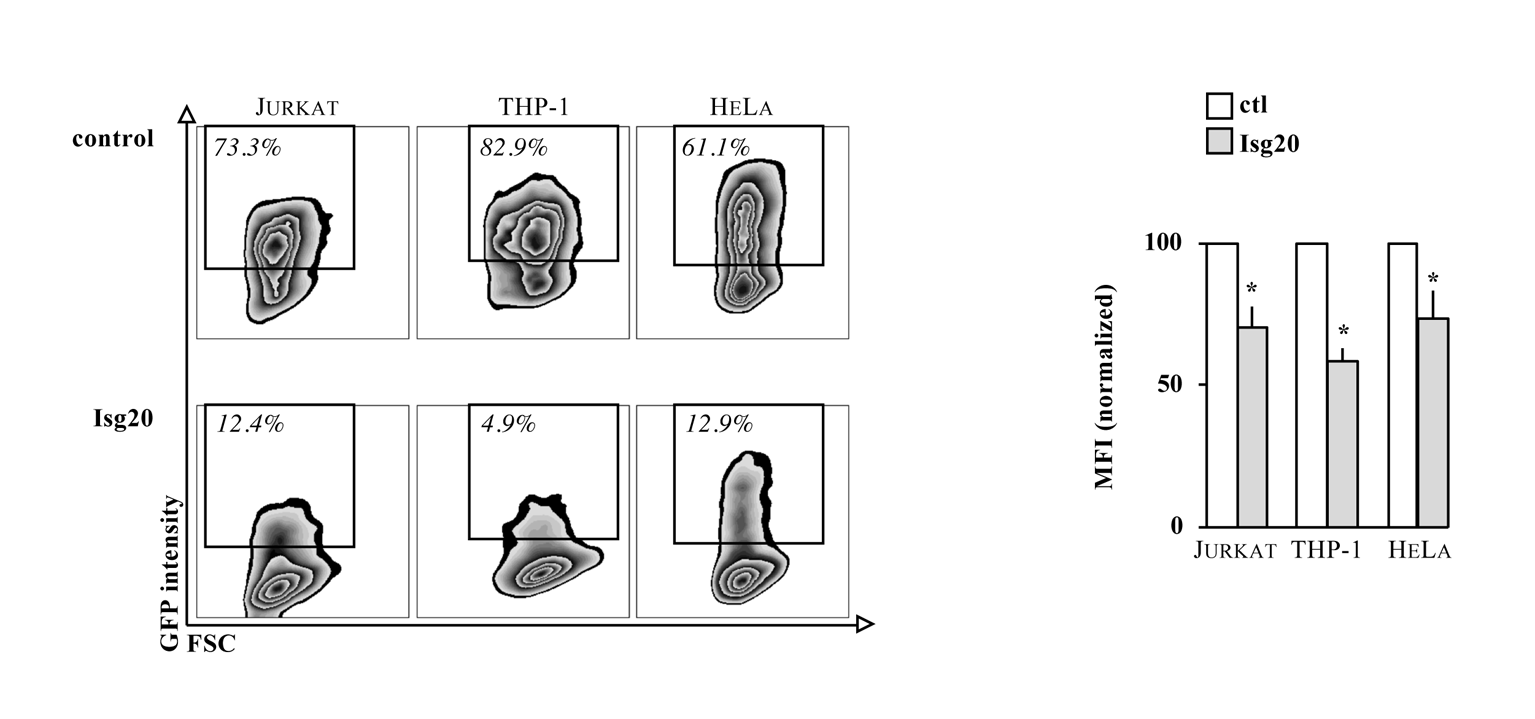

Supplement: S3 Fig — The figure depicts representative FACS panels obtained during VSV infection. The percentage of GFP-positive cells is displayed in each panel. The graph presents variations of the MFI in GFP-positive populations in the different conditions corresponding to the latest two points of the replication curves of Fig 1A. *, p≤0.05 according to a Student t test comparing control and ISG20 conditions. (TIF) [file ppat.1008093.s003.tif]

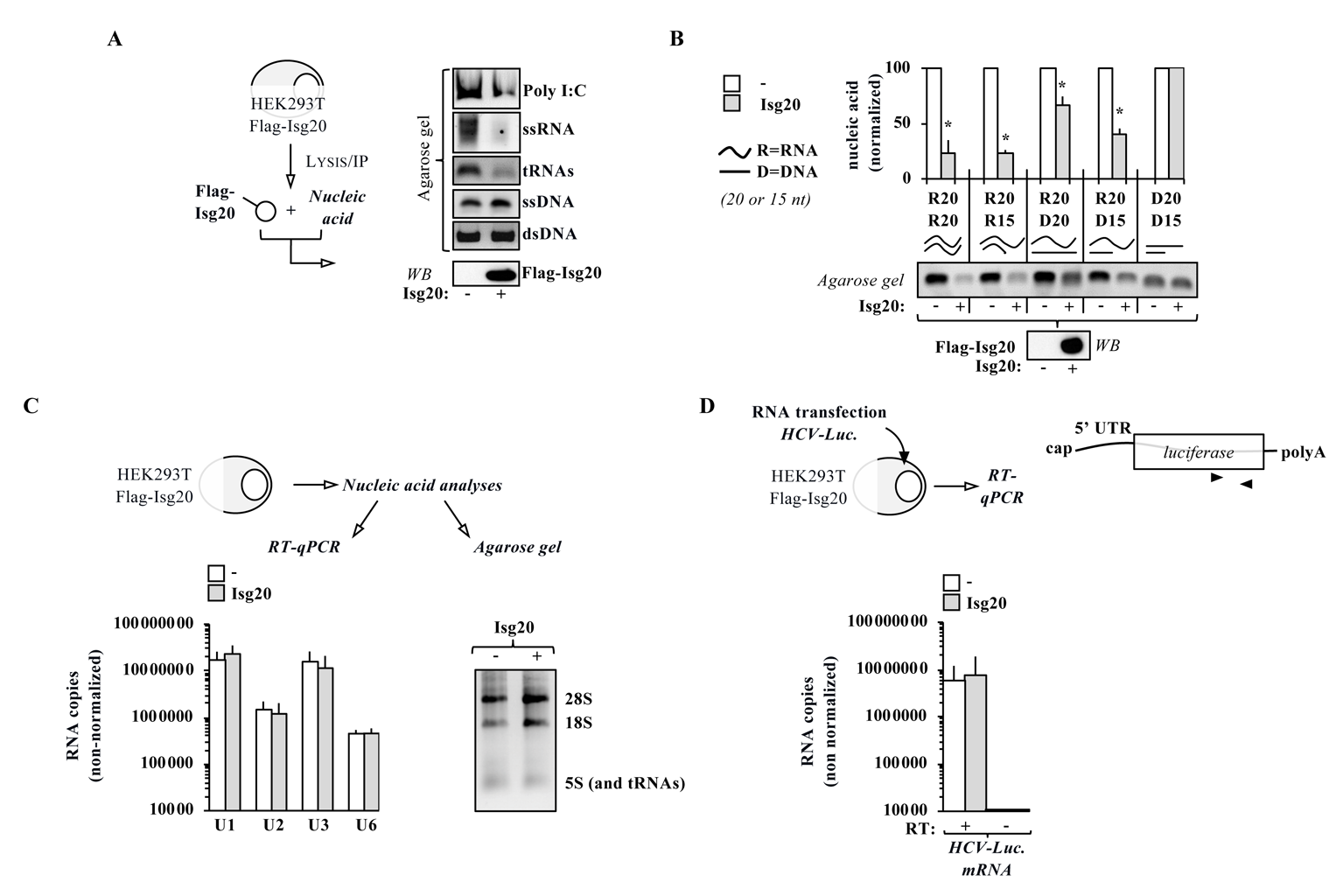

Supplement: S4 Fig — A and B) HEK293T cells transiently transfected in 10 cm plates with ISG20 coding DNA were lysed and ISG20 immunoprecipitated via anti-flag antibodies conjugated to agarose beads. After washing, beads bound material was incubated with the indicated nucleic acids (1 μg), prior to loading on agarose gels and densitometry quantification. Nucleic acids were as follows: ssRNA, in vitro transcribed single-stranded RNA, length of approximatively 1700 nucleotides; ssDNA, single-stranded DNA, oligonucleotide of 76 nucleotides; dsDNA, double stranded pcDNA3 plasmid (Invitrogen) linearized with PstI; poly I:C and yeast tRNAs, self-explicatory. In B, the indicated RNA and DNA forms were generated as described in the Methods section. C) HEK293T cells transiently transfected as above were directly lysed and the amount of small nuclear RNAs (U1, U2, U3 and U6) or of total RNA was evaluated by RT-qPCR and agarose migration, respectively. D) As in C but cells were also transfected with 200 ng of in vitro transcribed mRNA containing the 5’ UTR of the hepatitis C virus (HCV) and bearing the Firefly luciferase. Upon cell lysis the amount of transfected HCV-Luc mRNA was determined by RT-qPCR. A schematic representation of the RNA target and the position of the PCR amplicon is provided. The graphs present Means and SEM of independent experiments (n = 2 to 3 depending on target for B; n = 3 for C; n = 4 for D). The panels present typical results obtained. *, p≤ 0.05, following a Student t test. (TIF) [file ppat.1008093.s004.tif]

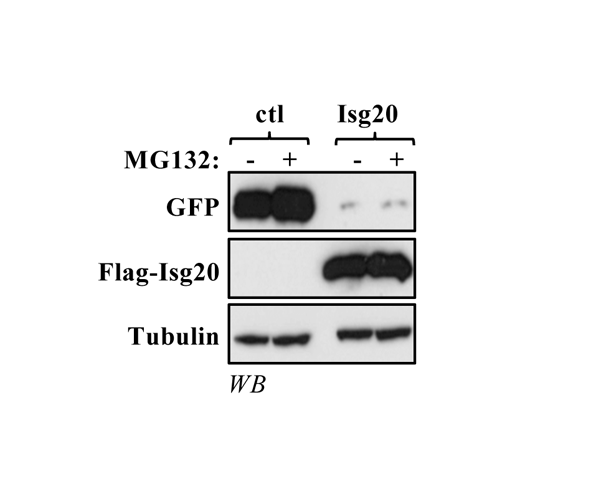

Supplement: S5 Fig — HEK293T cells were transiently transfected with a plasmid coding for GFP reporter and incubated with 10 μg/ml of MG132 (Sigma) overnight, prior to cell lysis and WB analysis. The panels present typical results obtained. (TIF) [file ppat.1008093.s005.tif]

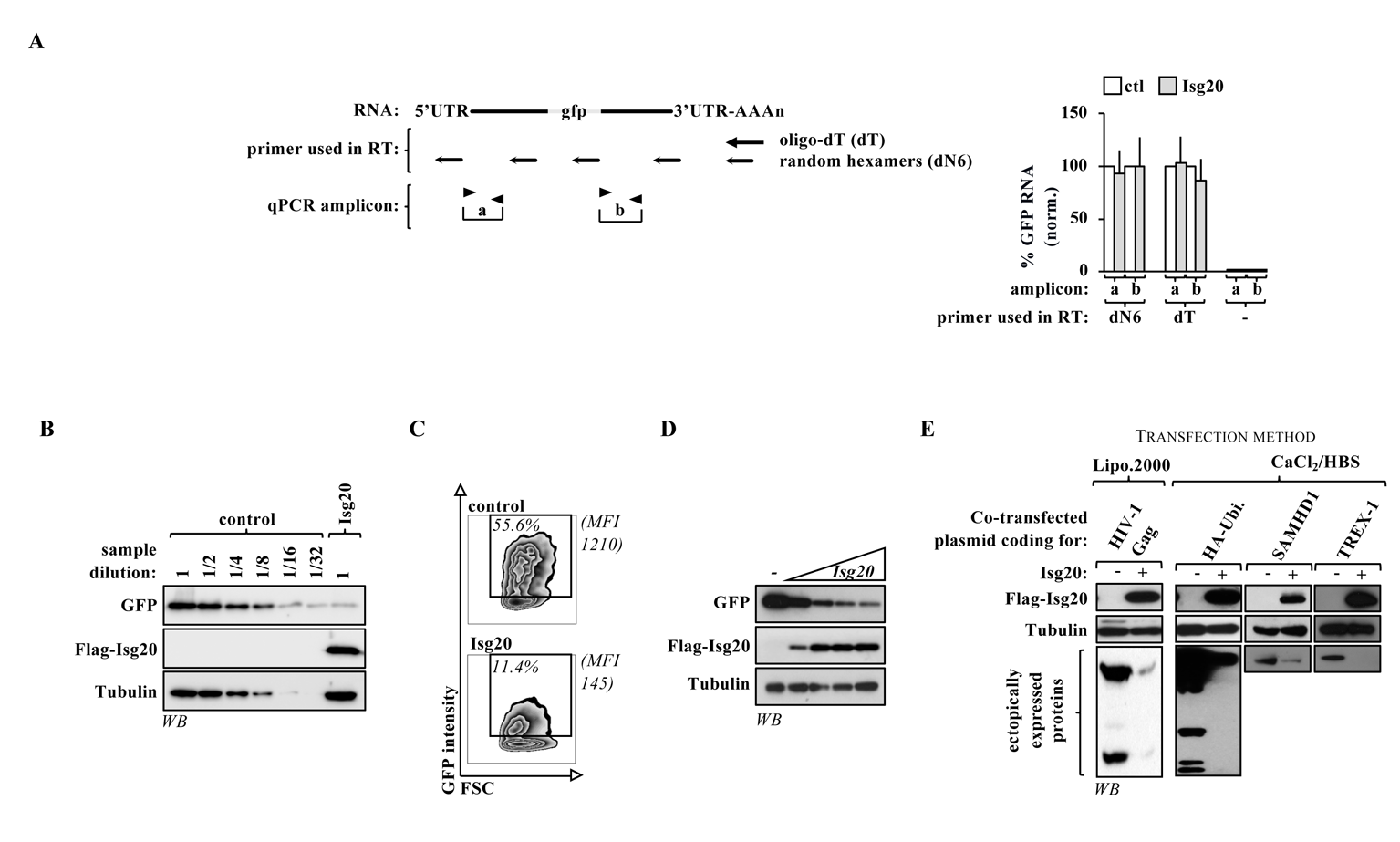

Supplement: S6 Fig — A) RNA obtained from HEK293T cells transiently transfected as in Fig 2B was reverse transcribed with either oligo-dT or random hexamers and then amplified with the indicated primers placed at different locations in the target GFP mRNA (the amplicon referred to as b is the one routinely used in the remaining figures). The graph presents results of 3 independent experiments. B) HEK293T cells transiently transfected with ISG20 and GFP coding plasmids (as in Fig 2B) were analyzed by WB twenty-four hours afterwards. To better appreciate the linearity and magnitude of the defect in GFP accumulation by WB, the control sample was serially diluted. C) As above, but cells were analyzed by flow cytometry to appreciate the decrease in the percentage of GFP-positive cells (displayed in the panels), as well as their MFI. D) HEK293T cells were transiently transfected with a fixed dose of GFP reporter and increasing concentrations of ISG20 followed by cell analysis by WB (from 1:1 to 1:8). E) HEK293T cells were transfected with DNAs coding for indicated proteins using either Calcium phosphate or lipofectamine-based DNA transfection, prior to WB analysis. (TIF) [file ppat.1008093.s006.tif]

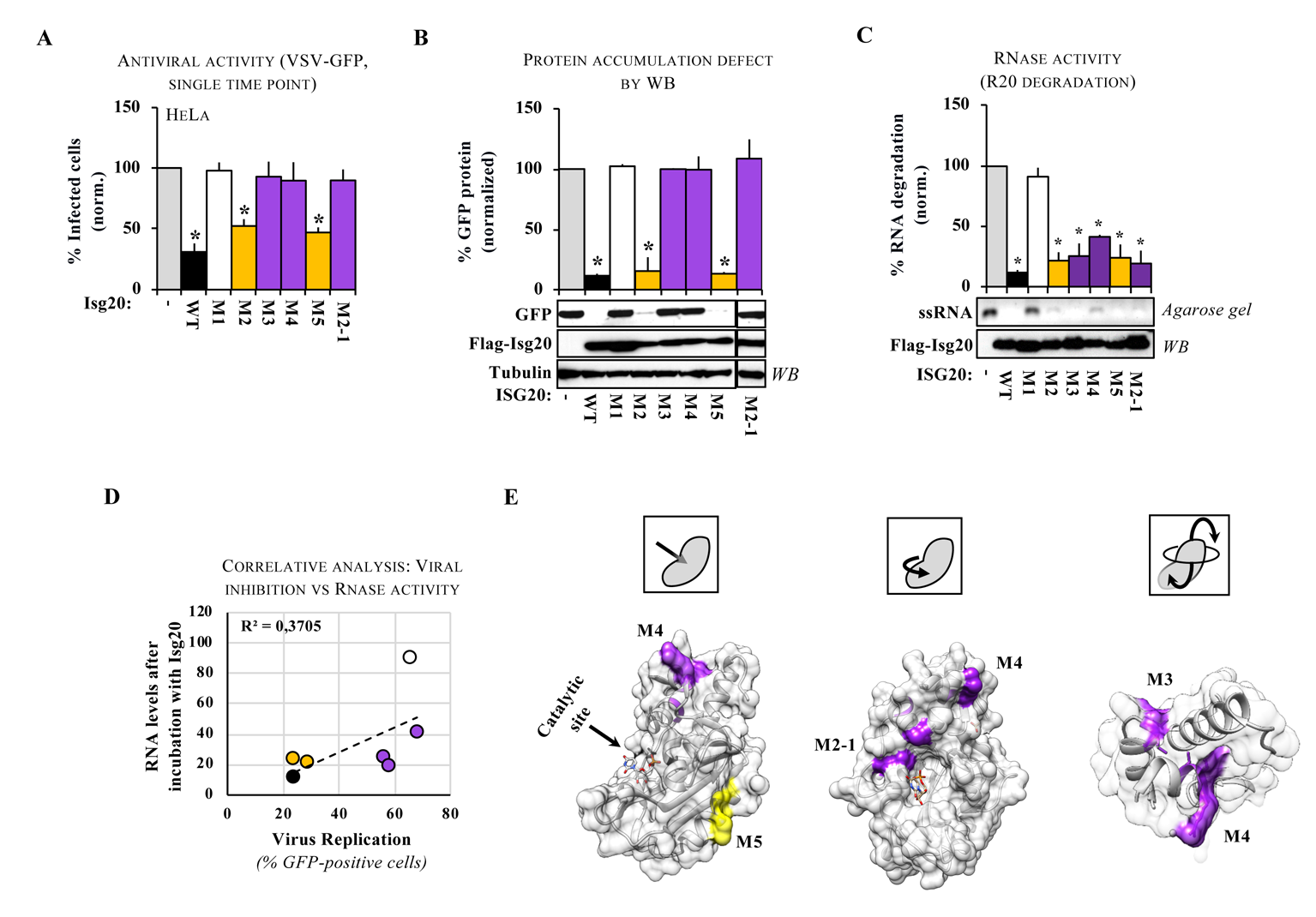

Supplement: S7 Fig — A) HeLa cells expressing the different ISG20 mutants were challenged with an MOI of 0.01 of VSV-GFP and the extent of viral infection was measured at a single time point, fifteen hours afterwards by flow cytometry. B) HEK293T cells were transfected with DNAs coding the different ISG20 mutants along with a GFP reporter. The amount of GFP reporter was then determined by densitometry after WB. C) The different ISG20 mutants were immunoprecipitated from transfected HEK293T cells and incubated with a single-stranded RNA oligo (R20). Upon migration on an agarose gel, the amount of intact RNAs were measured by densitometry. D) Correlative analysis between the RNAse and antiviral properties of individual ISG20 mutants. The graph present Means and SEM of four individual experiments (two for panel B). *, p≤ 0.05 following a Student t test. E) Spatial positioning of the indicated mutations on the crystal structure of ISG20. (TIF) [file ppat.1008093.s007.tif]

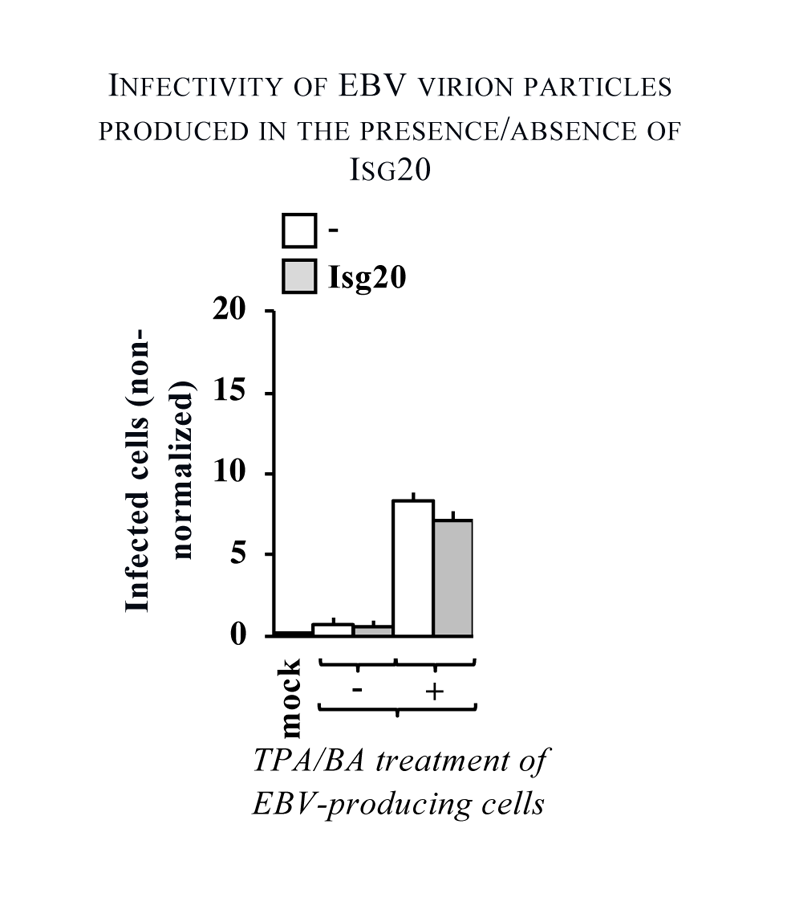

Supplement: S8 Fig — Hone cells containing a latent EBV genome bearing GFP were transfected with ISG20 and EBV reactivated from latency upon TPA/BA treatment as in the legend to Fig 2D. To determine whether ISG20 could affect the translation of the plethora of virion products required for the production of infectious virion particles, supernatants were syringe-filtered and virion infectivity was measured after challenge of target Raji cells and flow cytometry analysis three days later. (TIF) [file ppat.1008093.s008.tif]

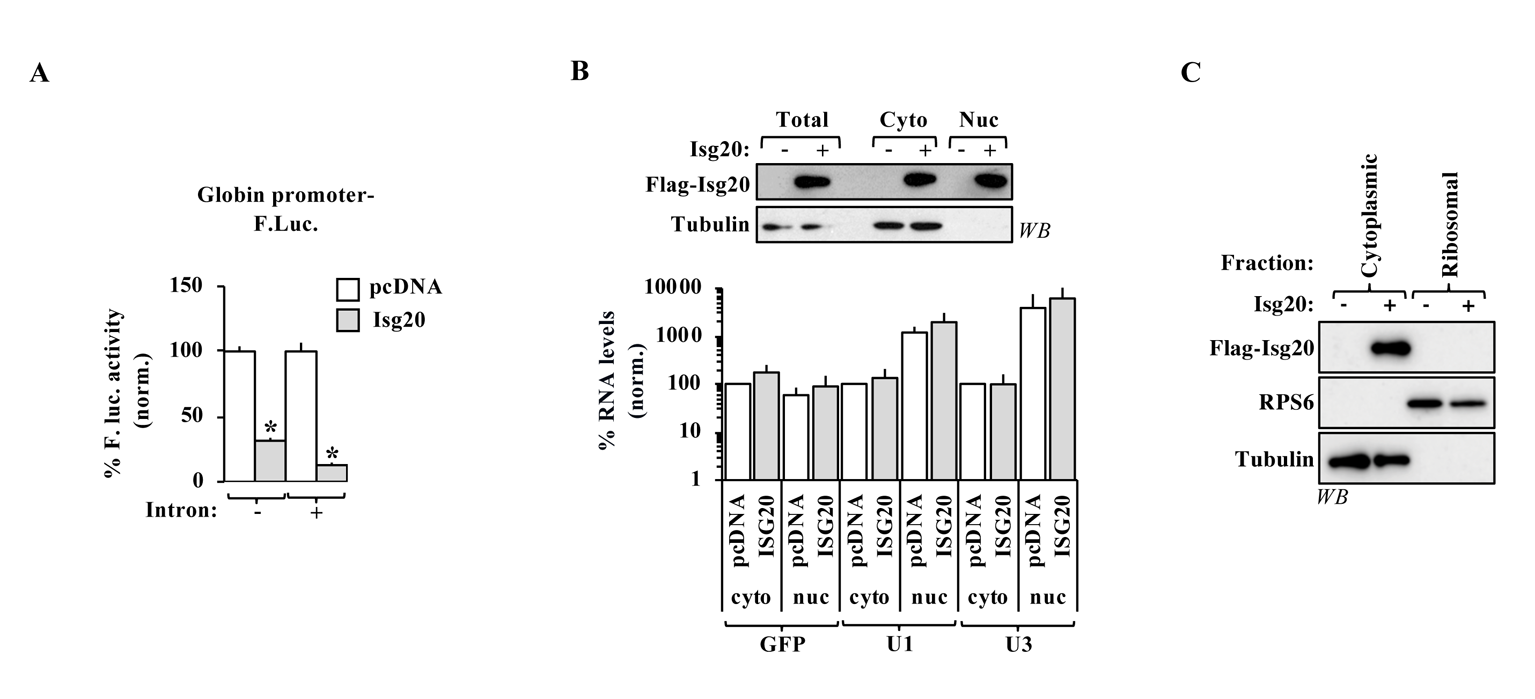

Supplement: S9 Fig — A) HEK293T cells were cotransfected with ISG20 along with two constructs coding for a Firefly luciferase reporter expressed from a globin promoter and containing or not an intron, prior to cell lysis and luciferase measurement. B) HEK293T cells were transiently transfected with ISG20 and GFP-coding plasmids and subsequently lysed to obtain nuclear and cytoplasmic fractions. Upon normalization of the two fractions by volume, samples were analyzed by WB and RT-qPCR (GFP). The distribution of two small nuclear RNAs known to be enriched in the nucleus (U1 and U3 by RT-qPCR) was also included as control for fractionation. C) HEK293T cells transfected with ISG20 were lysed, the ribosomal fraction purified, followed by WB analysis. The graphs present data obtained from two (Luc) and five (PCRs) independent experiments and panels present typical results. *, p≤ 0.05, following a Student t test. (TIF) [file ppat.1008093.s009.tif]

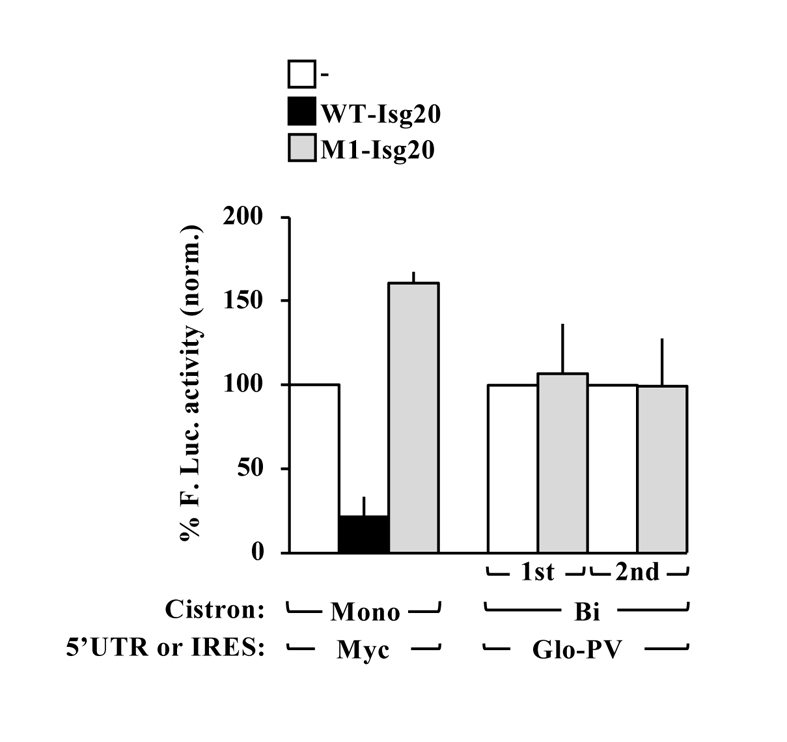

Supplement: S10 Fig — HEK293T cells were transfected with the indicated reporters along with WT or M1-ISG20 mutant prior to cell lysis and luciferase assay measurement. The graph presents data obtained from two and four independent experiments. Glo, globin; PV, polyomavirus; Myc, c-Myc. The nomenclature 1st and 2nd refers to the position of the cistron in bicistronic vectors. (TIF) [file ppat.1008093.s010.tif]

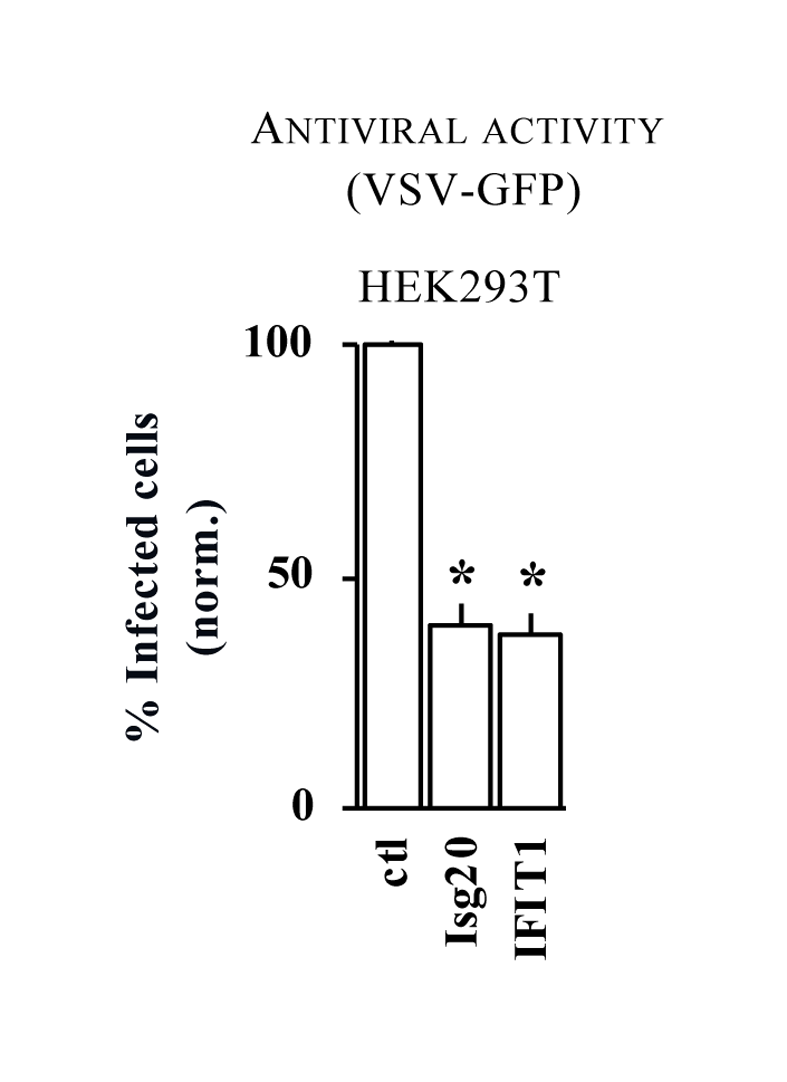

Supplement: S11 Fig — HEK293T cells transfected with plasmids coding the above-mentioned proteins were challenged twenty-four hours afterwards with VSV-GFP at an MOI of 0.01 prior to flow cytometry analyses twenty four hours later. The graph presents results obtained with three independent experiments. *, p≤ 0.05, following a Student t test between control cells and the indicated condition. (TIF) [file ppat.1008093.s011.tif]

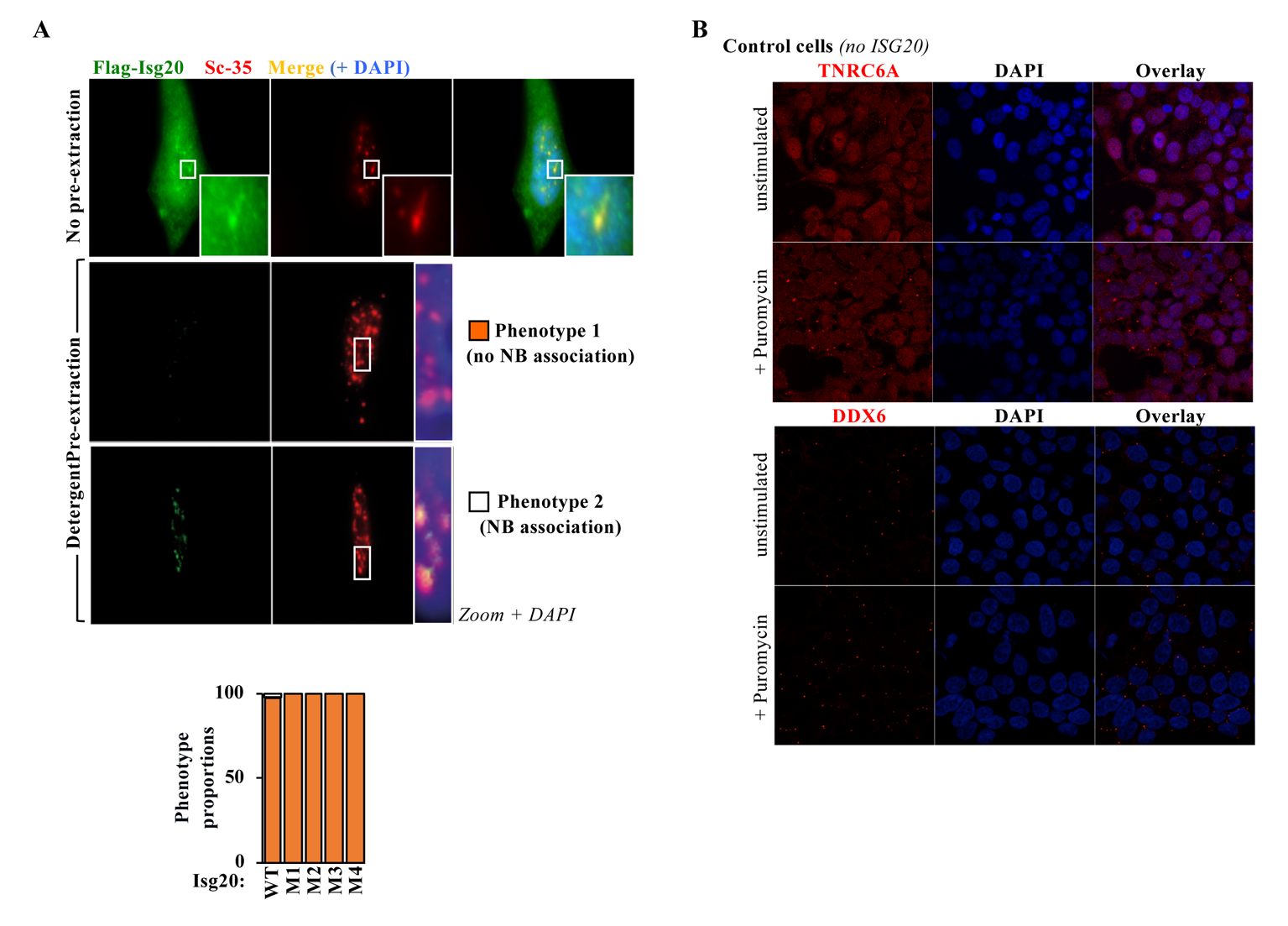

Supplement: S12 Fig — A) HEK293T cells were transiently transfected in duplicate with plasmids coding ISG20 (routine transfection rates ≥80%). Twenty-four hours afterwards, one aliquot was immediately fixed while the second was first permeabilized with detergent prior to fixation. Both were then similarly processed and analyzed by confocal microscopy using antibodies specific for ISG20 (Flag), as well as for the nuclear speckles marker SC35. This procedure is commonly used to study nuclear bodies and associated proteins that resist detergent extraction prior to fixation. Representative pictures and relative distributions are show here (>80 cells scored per condition). B) Control cells were analyzed by confocal microscopy with the endogenous P bodies markers TNRC6A and DDX6 (ISG20-expressing cells and a portion of the overlay of control cells depicted here are presented in Fig 5A). (TIF) [file ppat.1008093.s012.tif]

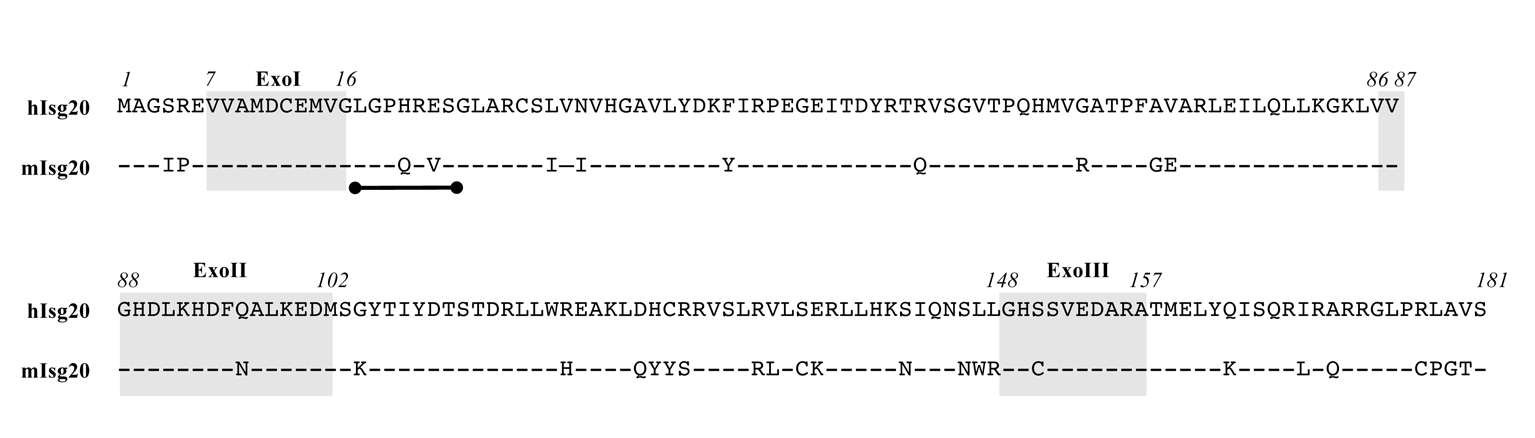

Supplement: S13 Fig — Amino acids alignment of human and murine ISG20s. The black bar presents the CRISPR/Cas9 target sequence used. Shaded boxes indicate the position of the exonuclease domains I to III. (TIF) [file ppat.1008093.s013.tif]
